# CTNNBL1 facilitates the association of CWC15 with CDC5L and is required to maintain the abundance of the Prp19 spliceosomal complex

**DOI:** 10.1093/nar/gkv643

**Published:** 2015-06-29

**Authors:** Febe van Maldegem, Sarah Maslen, Christopher M. Johnson, Anita Chandra, Karuna Ganesh, Mark Skehel, Cristina Rada

**Affiliations:** 1 MRC Laboratory of Molecular Biology, Cambridge, CB2 0QH, UK; 2 Department of Medicine and Cancer Biology & Genetics Program, Memorial Sloan Kettering Cancer Center, New York, NY 10065, USA

## Abstract

In order to catalyse the splicing of messenger RNA, multiple proteins and RNA components associate and dissociate in a dynamic highly choreographed process. The Prp19 complex is a conserved essential part of the splicing machinery thought to facilitate the conformational changes the spliceosome undergoes during catalysis. Dynamic protein interactions often involve highly disordered regions that are difficult to study by structural methods. Using amine crosslinking and hydrogen–deuterium exchange coupled to mass spectrometry, we describe the architecture of the Prp19 sub-complex that contains CTNNBL1. Deficiency in CTNNBL1 leads to delayed initiation of cell division and embryonic lethality. Here we show that *in vitro* CTNNBL1 enhances the association of CWC15 and CDC5L, both core Prp19 complex proteins and identify an overlap in the region of CDC5L that binds either CTNNBL1 or CWC15 suggesting the two proteins might exchange places in the complex. Furthermore, *in vivo*, CTNNBL1 is required to maintain normal levels of the Prp19 complex and to facilitate the interaction of CWC15 with CDC5L. Our results identify a chaperone function for CTNNBL1 within the essential Prp19 complex, a function required to maintain the integrity of the complex and to support efficient splicing.

## INTRODUCTION

The Prp19 complex is an essential multi protein component associated with the splicing machinery that consists of a core of four conserved proteins (1), in humans PRPF19, CDC5L, PLRG1 and SPF27 (the product of the *BCAS2* gene), and a sub-complex that includes CTNNBL1 and CWC15, as well as the chaperone protein HSP7C (encoded by the *HSPA8* gene) (2). As part of a bigger complex of more than 30 associated proteins (3), the Prp19 complex aids the progression of the core snRNP components through several steps of catalysis and recycling and is therefore essential for splicing (4). The core complex has remarkable conservation of function, being essential in both *Saccharomyces cerevisiae* and *Schizosaccharomyces pombe*—known as the NineTeen Complex or NTC (5). The precise role of each of the components of the core complex is still uncertain (6), although as a whole it is thought to play a role facilitating the dynamic conformation rearrangements that take place during the maturation of the spliceosome (7,8).

PRPF19 (also known as PSO4 (9)) consists of a U-box domain with E3 ligase activity (10), a coiled-coil region that mediates its tetramerization and interaction with CDC5L and SPF27, as well as seven WD interaction domains that link it to other components of the spliceosome. The integrity of the U-box and the ability to act as a tetramer are essential for its function as part of the Prp19 complex (11). In addition to splicing, PRPF19 has been linked to other RNA processing functions, such as facilitating elongation and transcription coupled repair (12,13) or mRNA quality control and export in yeast (14). PRPF19 was initially identified in a screen for mutant genes conferring sensitivity to DNA damage by crosslinking agents, and has been shown to be involved in the activation of the ATR (Ataxia telangiectasia and Rad3 related) signalling cascade in response to DNA damage and replication stress (15,16), as well as in the control of mitotic spindle assembly (17). It is clear that PRPF19 has multiple roles in addition to its function as part of the spliceosome (reviewed in (2)).

CDC5L was originally identified in a cell cycle screen in yeast and has been implicated in cell cycle checkpoint function and nucleic acid binding, linking the enzymatic activity of PRPF19 to U2-U6 RNPs and other potential targets (18,19). It is still unclear whether it functions outside the Prp19 complex independent of PRPF19, at least in mammalian cells, as CDC5L has also been implicated in ATR signalling (15,20). PLRG1 contains multiple WD motifs that mediate protein/protein interactions through which it binds CDC5L and links it to other core components (21), although it is likely that additional cellular roles outside the Prp19 complex make it essential (22). SPF27 was initially identified as a cDNA amplified in human breast cancers (BCAS2) (23), and is a unique protein domain of unknown function. Although conserved, the SPF27 homologue is not essential in *S. cerevisiae* unlike in *S. pombe* (24), and is considered a scaffold component in the Prp19 complex.

CTNNBL1 is considered a peripheral component of the Prp19 complex since it has not been found consistently associated with the complex across species (5,25) and does not remain part of the complex throughout the spliceosome maturation (26). CTNNBL1 is an ARM domain protein with structural similarity to karyopherin (27) and known to bind nuclear localization signals, including those of CDC5L, PRP31 and non spliceosome proteins such as the antibody diversification enzyme AID (28,29). CTNNBL1 and CWC15 are also present as a stable heterodimer outside the Prp19 complex (30), but unlike CTNNBL1, CWC15 is essential for cell viability and has no assigned structural domains or function. Even less is known about the role of HSP7C, which is present only in a minority of Prp19 complexes (31), but its generic function as a chaperone indicates that it might be transiently required to sense misfolding associated with the complex.

Molecular interactions in highly dynamic complexes such as the spliceosome often involve intrinsically disordered regions that are not amenable to structural analysis (32). Similarly, structural information for CTNNBL1 has not aided mutagenesis-based mapping of its interactions with other proteins (33). In order to understand the interactions of the CTNNBL1/CWC15 sub-complex in relation to the core Prp19 complex, we have combined biochemical and hydrogen–deuterium exchange mass spectrometry (HDX-MS) methods to identify the associations of CTNNBL1 with the Prp19 complex proteins *in vitro*, bringing us a step closer to understanding the dynamics of the complex *in vivo*, where CTNNBL1 appears to be required to maintain the integrity of the Prp19 complex through facilitating association of CWC15 with CDC5L and thereby support efficient function of the complex in splicing.

## MATERIALS AND METHODS

### Recombinant protein expression and purification

Plasmids for CTNNBL1, CWC15 and CDC5L and deletion mutants (see Supplementary Methods Table S1) were transformed into BL21 Rosetta (DE3)pLysS cells, grown to a density of 0.8–1.0 under ampicillin and chloramphenicol selection, and protein expression induced with 1 mM IPTG overnight at 18°C. Cells were harvested and lysed by sonication. Cleared lysates of His-CTNNBL1, GST-CWC15 and MBP-CDC5L were incubated with Ni-NTA (Qiagen), Glutathione-Sepharose 4B (GE Life Sciences) or Amylose resin (New England Biolabs) respectively, for 1 h at 4°C, washed and eluted using 1M imidazole, 10 mM reduced glutathione or 50 mM maltose, respectively. For gel filtration, crosslinking and HDX, the tags of CWC15 and CDC5L were cleaved off by HRV 3C and Tobacco Etch Virus (TEV) proteases respectively, followed by a second purification step on a 6 ml Resource Q ion exchange column in the case of CTNNBL1 and CWC15, or a Resource S ion exchange column in the case of CDC5L using an AKTA Prime Plus FPLC system (GE Lifesciences). Elution was performed against a gradient of buffers A and B (all buffers are listed in Supplementary Methods).

### Pull downs

For recombinant protein pull down assays 10–20 μg of each purified protein in pull down buffer was combined and incubated with 25 μl affinity resin for 1 h at 4°C under constant mixing. The resin was washed four times with the same buffer and the complexes were eluted off the beads using spin columns. The eluates were clarified by centrifugation 10 min 21 000 g, supernatants and input samples were separated by SDS-PAGE and visualized using Instant Blue (Expedeon).

### Crosslinking-MS and HDX-MS

(Full details of experimental procedures are described in Supplementary Methods). Briefly, purified recombinant human proteins with or without proteolytic tag removal were crosslinked with BS3 d0/d4 in crosslinking buffer, after which SDS-PAGE gel bands were further processed for MS analysis. For HDX-MS, protein complexes were pre-formed and dialysed in HDX exchange buffer without D_2_O, after which exchange in presence of 80% D_2_O was allowed for 3, 30, 300 and 3000 s before quenching by addition of quench buffer. Samples were snap frozen and analyzed by MS. The quenched protein samples were rapidly thawed and subjected to proteolytic cleavage by pepsin followed by reversed phase High-performance liquid chromatography (HPLC) separation essentially as previously described (34). Mass analysis of the peptide centroids was performed as described previously, using the software HD-Examiner (Sierra Analytics) (34).

### Data analysis of HDX

%HDX per peptide was defined as the number of deuterium atoms divided by the maximum number of hydrogen atoms theoretically available for exchange on the peptide (#D). Differences in %HDX were averaged for each time point and the maximum difference over any of the time points was defined as ΔHDX. The significant threshold used to call the interaction sites was defined as >10% average exchange difference (ΔHDX) with t-test *P* < 0.05 (unpaired, two-tail) and a minimum signal difference of at least 0.5 Da.

### Mouse B cell isolation

Splenic B cells from *CD19-Cre^+/−^ Ctnnbl1^−/flx^* mice (35) were MACS (Miltenyi Biotec) sorted for CD43 negative cells, following the manufacturer's protocol. *CD19-Cre^+/−^ Ctnnbl1^+/flx^* littermates were used as controls. The isolated resting B cells, typically ∼95% pure B cells, were counted and either washed with phosphate buffered saline (PBS), pelleted and snap frozen or cultured in complete RPMI (cRPMI: 10% FCS, penicillin, streptamycin, 50 μM 2-Mercaptoethanol, 0.1% BSA, 25 μg/ml lipopolysaccharide (LPS) (Sigma-Aldrich) and 25 ng/ml recombinant mouse IL-4 (R&D systems)). Mice were generated and maintained under UK Home Office regulations.

### Immunofluorescence

Freshly isolated resting B cells were attached to Poly-L-lysine (Sigma Aldrich) coated glass slides and fixed either in ice-cold methanol (−20°C) for 15 min and allowed to dry or in 4% paraformaldehyde for 20 min. After 30 min in 1%BSA/1% goat serum, 0.1% Triton-X100 in PBS, slides were incubated for 1 h with CDC5L (1:100, Abcam ab31779), PRPF19 (1:50, Abcam ab27692) rabbit antisera in in PBS, 0.1%BSA and 0.01% T-X100, washed and incubated with goat-anti-rabbit conjugated to Alexa Fluor 488 (1:100, Life Technologies), washed and mounted using Vectashield/DAPI (Vector Laboratories). Fluorescent signals were recorded with Radiance 2100 confocal microscope (Bio-Rad Laboratories) with a Plan Apo 60×/1.40 NA oil immersion lens (Nikon) using LaserSharp 2000 acquisition software (Bio-Rad Laboratories) (400x final magnification) or a Zeiss LSM780 (63x magnification) and quantified using ImageJ64.

### Retroviral transduction

HEK293T cells were co-transfected using Novagen GeneJuice (Merck Millipore) with retroviral vectors pEco and pMXiG (36) or pMXiG-CWC15. Supernatant harvested after 2–3 days was used to infect B cells in Retronectin (Takara)-coated plates 24 h post activation in the presence of 5 μg/ml Polybrene (Merck Millipore). After centrifugation at 650 g for 45 min at 32°C and overnight culture, cells were transferred to new tissue culture plates in cRPMI.

### siRNA transfections

Human osteosarcoma cell line U2OS was transfected with 100 nM of CWC15 or CDC5L ON-TARGETplus siRNA SMARTpools or a non-targeting siRNA pool as control (Dharmacon) using lipofectamine RNAiMAX transfection reagent according to manufacturer's protocol (Life Technologies) on two consecutive days. Cells were lysed on day three in supplemented triton lysis buffer and analysed by western blotting as described for B cells below.

### Immunoprecipitations and westerns

For immunoprecipitations, 2 × 10^6^ B cells per sample were lysed in triton lysis buffer supplemented with benzonase, lysolecithin, EDTA-free protease inhibitors (Roche) and PhosSTOP (Roche) and rotated for 30 min at 4°C. Clarified lysates (after 10 min centrifugation at 21 000 g) were either used directly for assessing protein abundance or were incubated 1 h at 4°C under constant mixing and addition of 25 μl anti-HA resin (Roche). The resin was washed three times with the same buffer and proteins boiled off the beads in sample buffer. To assess protein abundance 0.5 × 10^6^ B cells per sample were lysed as before. The proteins were visualized by western blotting using antibodies against HA (3F10, Roche), CDC5L (BD612362, BD Biosciences), CTNNBL1 (pab19409, Abnova), CWC15 (25293–1-AP, Proteintech), PRPF19, PRPF8, PLRG1, Lamin B1, β-tubulin and β-actin (ab27692, ab79237, ab86050, ab16048, ab6046, ab8224, from Abcam).

### Quantitative PCR

2 × 10^6^ B cells were either frozen immediately after isolation from spleen as described above or taken into culture in complete RPMI for 5 h before washing in PBS, pelleting and snap-freezing. RNA was extracted from the cell pellets using the PureLink RNA purification kit (Ambion) combined with on-column PureLink DNase treatment. cDNA was generated using random primers and High Capacity cDNA Reverse Transcription Kit (Applied Biosystems). Control reactions (minus reverse transcriptase) and sample cDNA was diluted and used as input in qPCR reactions with Power Sybr Green PCR Master Mix (Applied Biosystems) and the primers listed in Supplementary Methods Table S2. Reactions were run on a ViiA7 Real-Time PCR system (Life Technologies). cycle threshold (CT) values obtained for unspliced transcripts were corrected against background by subtracting values of –RT controls. For each transcript, spliced/unspliced ratios were corrected for the efficiency of the PCR based on the slope of a standard curve consisting of a serially diluted cDNA pooled from primary B cells RNA.

See Supplementary Table S3 for nomenclature of the human and mouse Prp19 complex proteins and species homologues. Statistic analyses were performed using Prism 6 (GraphPad Software, La Jolla California, USA).

## RESULTS

### Enhanced stability of a CTNNBL1, CDC5L and CWC15 trimeric complex

**Figure 1.**
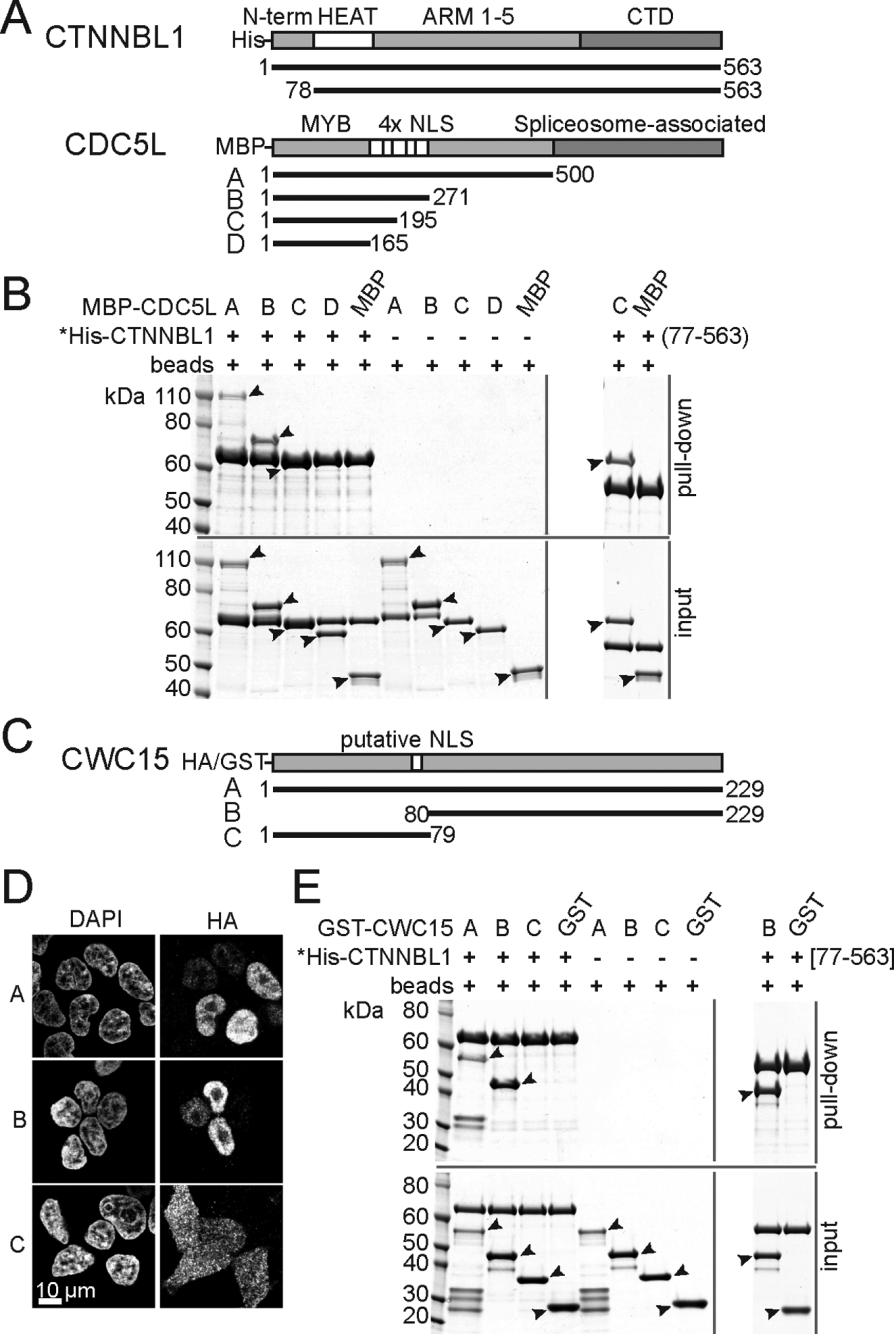
CTNNBL1 interacts directly with the N-terminal region of CDC5L and the C-terminus of CWC15. (**A**) Linear structure of human CTNNBL1, CDC5L and truncation mutants. Known domains, tags for pull down and aa numbering are indicated. (**B**) Pull down of full-length recombinant His-CTNNBL1 on Ni-NTA resin in the presence of recombinant MBP-CDC5L fragments or MBP only controls. The MBP proteins are not present in the pull down in the absence of His-CTNNBL1 showing the specificity of the pull down. The size of the MBP-CDC5L fragment C overlaps with the size of full-length His-CTNNBL1 but the binding can be detected by pull down using the His-CTNNBL1[77–563] fragment as shown on the right panel. Asterisks indicate the bait used for the pull downs with the lower panels in each indicating the input proteins. Molecular size markers (kDa) are shown. Filled arrows next to the bands identify the MBP fusions. (**C**) Linear structure of human CWC15 and truncation mutants. The predicted nuclear localization signal NLS, tags and aa numbering are indicated. (**D**) Confocal immunofluorescence of HeLa cells transfected with CWC15 HA fusion proteins A, B and C stained with DAPI on the left panel and anti-HA antibodies on the right. (**E**) Pull down of His-CTNNBL1 as in B, in the presence of recombinant CWC15 GST fusions. The panel on the right shows the interaction of the C-terminal fragment of CWC15 with the truncated His-CTNNBL1[77–563] by pull down of the GST-CWC15 fragment B compared to GST alone as a control. As in (B), the asterisks indicate the bait of the pull down with the lower panels showing the input proteins and the filled arrows identifying the GST fusion fragments. Molecular size markers (kDa) are shown.

Biochemical purification of the Prp19 complex has defined the overall conformation of the core proteins, suggesting that CTNNBL1 interacts with the complex via CDC5L (30). We have previously shown that CTNNBL1 is a novel nuclear localization signal (NLS) binding ARM domain protein that specifically interacts with the NLS region of CDC5L (28). Here we use various recombinant mutants of CDC5L (as maltose binding protein (MBP) fusions) and full-length recombinant His tagged CTNNBL1 in pull downs (Figure 1A) to define the minimal regions involved in binding between the two proteins. As shown in Figure 1B, fragments of CDC5L that include the NLS region do interact directly with CTNNBL1, even fragment C just containing NLS1–2, indicating that the previously identified NLS3 is sufficient but not essential for the interaction. The unstructured N-terminal domain of CTNNBL1 (33,37,38) is not required for binding as the pull down can be recapitulated using the N-terminal truncation mutant of CTNNBL1[77–563]. In agreement with previous mutagenesis results (33), we found that the CDC5L[1–195] fragment retained its interaction with CTNNBL1 mutants of the putative NLS binding region predicted by structural homology to karyopherin alpha (Supplementary Figure S1).

Pull down experiments in HeLa cells have shown CTNNBL1 in a stable binary complex with CWC15, a small 27kDa protein and an essential component of the Prp19 complex (30). We therefore tested whether CWC15 might interact with CTNNBL1 via an NLS motif. Unexpectedly, the N-terminal fragment of human CWC15, which contains a predicted NLS sequence aa 60–76 (Figure 1C), did not localize to the nucleus (Figure 1D) while the C-terminal fragment did, suggesting it might also mediate the interaction with CTNNBL1. Indeed, recombinant His-tagged CTNNBL1 failed to bring down the GST tagged CWC15[1–79] fragment, whereas full-length GST-CWC15 and GST-CWC15[80–229] were efficiently recovered (Figure 1E). This result also shows that the interaction between the two proteins is direct. As was the case for CDC5L, the N-terminus of CTNNBL1[1–76] was not involved in binding to CWC15 (Figure 1E, right). These results suggest that the functional NLS of CWC15 is located in the C-terminal fragment of the protein, the fragment that also mediates the interaction with CTNNBL1.

**Figure 2.**
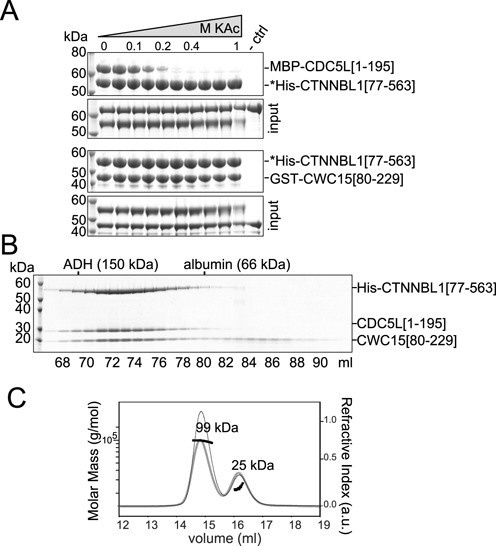
CTNNBL1 interacts differently with CDC5L or CWC15 but all three form a stable sub-complex. (**A**) Pull downs of His-CTNNBL1[77–563] on Ni-NTA in the presence of increasing potassium acetate (KAc) concentrations (0, 0.05, 0.1, 0.15, 0.2, 0.3, 0.4, 0.6, 0.8 and 1 M) and either MBP-CDC5L[1–195] on the top or GST-CWC15[80–229] bottom. A control pull down (-ctrl) using beads was performed in 0.11 M KAc. Asterisks indicate bait of pull downs and size markers (kDa) are shown. For each experiment, the proteins used for pull down are labelled input and shown below. (**B**) SDS-PAGE analysis of recombinant human His-CTNNBL1[77–563], CDC5L[1–195] and CWC15[80–229] protein mix after Superdex-200 gel filtration. The elution volume (ml) of the fractions analysed and the fractions eluting molecular weight markers of known size (alcohol de-hydrogenase (ADH) and albumin) are indicated. (**C**) As in B, a protein mixture of purified His-CTNNBL1[77–563], CDC5L[1–195] and CWC15[80–229] analysed by SEC-MALS. The grey curve shows the refractive index (arbitrary units) against the elution volume (ml) (right y-axis). The horizontal black lines indicate the molar mass distribution in g/mol (left y-axis). The calculated molar mass for the two observed peaks are indicated, with a large peak corresponding to a protein complex of 99 kDa and a second small protein complex of 25 kDa which corresponds to the predicted molecular mass of monomeric CWC15[80–229] fragment. Based on the UV absorbance of the eluted main peak (thin line) a 102 kDa protein complex with a 1:1:1 stoichiometry could be inferred based on the extinction coefficient of the three protein fragments in the mix.

*In vitro*, binding of CTNNBL1 to CDC5L[NLS3] is affected by salt concentrations, suggesting the interaction is mediated by electrostatic forces (33) whereas *in vivo*, biochemical purification of human Prp19 complexes only retained CTNNBL1 under low salt conditions (1,30). To test the nature of the interactions of CTNNBL1 with the CDC5L and CWC15 fragments, we performed pull downs under different ionic strength; and while the CTNNBL1-CDC5L interaction was lost at potassium acetate (KAc) concentrations above 0.15 M, binding was retained for CWC15 up to 1M KAc (Figure 2A). We then questioned whether CTNNBL1[77–563] could bind CDC5L and CWC15 at the same time, as was proposed by Grote *et al*. (30). After removal of their tags, recombinant CWC15 and CDC5L were allowed to form a complex with CTNNBL1. Separation of the mix on a Superdex200 column confirmed a stable complex under gel filtration conditions, with the three proteins co-migrating as a high molecular weight complex (Figure 2B). Size exclusion chromatography coupled with multi-angle light scattering (SEC-MALS) revealed a monodisperse complex with a mass of 99 kDa, equivalent to a 1:1:1 stoichiometry for each protein (Figure 2C). Thus binding of CTNNBL1 to its two partners is dissimilar; with CDC5L mostly binding through electrostatic interactions, mediated through charged residues in the NLS-region, while binding to CWC15 is likely through hydrophobic interactions. Nonetheless, the three proteins adopt a stable conformation *in vitro*, with one copy of each protein present in the complex.

**Figure 3.**
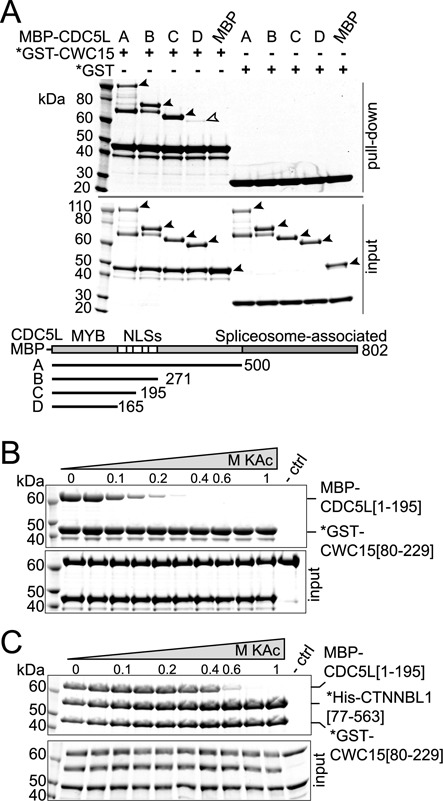
CTNNBL1 stabilizes binding of CDC5L and CWC15 at high salt. (**A**) Pull down on glutathione Sepharose beads of GST-CWC15[80–229] in the presence of CDC5L MBP fusion fragments depicted or MBP alone. Input proteins are shown in the lower panel. The open arrow denotes fragment D of CDC5L, corresponding to the N-terminal region that includes the MYB domain. Pull down of GST-CWC15[80–229] in the presence of (**B**) MBP-CDC5L[1–195] or (**C**) both MBP-CDC5L[1–195] and His-CTNNBL1[77–563] at increasing potassium acetate (KAc) concentrations (as in Figure 2A). Pull down of control beads in 0.11 M KAc (-ctrl). Asterisks denote the bait in the pull downs. The proteins in the input are shown on the lower panel. Size markers are indicated (kDa).

In order to test whether the complex is stable as a result of a three-way interaction or it exists in a linear configuration, we performed pull downs of CWC15[80–229] and truncations of CDC5L in the absence of CTNNBL1. The results show that CWC15 and CDC5L can interact directly and do so through the NLS region of CDC5L (Figure 3A). A faint band could be detected in the pull down for the smallest fragment of CDC5L (Figure 3A, open arrow), which was absent in the pull downs with CTNNBL1 (see Figure 1B), suggested that the N-terminus of CDC5L might also contribute to the interaction. A single CDC5L[NLS3] region was unable to pull down CWC15 (Supplementary Figure S2) in contrast to previous observations for CTNNBL1 (28), again suggesting that the binding site on CDC5L might require additional surface to retain CWC15. Furthermore, pull downs over a range of salt concentrations showed that the CWC15[80–229]/CDC5L[1–195] interaction was only stable at low ionic strengths (Figure 3B) raising the question of whether such weak interactions at physiological salt concentrations could be relevant. We thus repeated the salt-range pull downs, this time testing interaction of all three proteins as a complex (Figure 3C). Under these conditions, CDC5L only dissociated from the complex at concentrations of 0.6 mM KAc or higher, demonstrating that the stability of the interactions is greatly enhanced when all three proteins are present in the complex. Thus it is likely that the three proteins in the complex adopt a three-way conformation that promotes that stability of the interactions.

### Architecture of the CTNNBL1, CDC5L and CWC15 trimeric complex

As extensive mutagenesis of CTNNBL1 had failed to identify the site of binding for the CDC5L NLS region (33) or the CDC5L[1–195] fragment (Supplementary Figure S1), we used a Bis (sulfosuccinimidyl) suberate crosslinking reagent mix combined with mass spectrometry to identify regions in physical proximity within binary and ternary complexes of the three protein fragments, His-CTNNBL1[77–563], CDC5L[1–195] and CWC15[80–229] (Supplementary Figure S3). The resulting peptide map revealed extensive crosslinks in the CWC15/CTNNBL1 complex, for peptides encompassing the C-terminus of CWC15 (including K204, K205 and K207) and the first helix of the pre-ARM domain of CTNNBL1 (K84, K91 and K95) (Supplementary Figure S4A). In contrast, only a few crosslinks appeared between CDC5L and CTNNBL1 or CWC15 (Supplementary Figure S4B and C). Peptides corresponding to the NLS-region of CDC5L were overall underrepresented in the trypsin digest due to an abundance of arginines and lysines (Supplementary Figure S4D), and a similar effect was seen for the C-terminal lysine-rich fragment of CWC15. Despite these limitations, crosslinks were detected from NLS1 to loop 2 of ARM5 at the side of the solenoid and to the N-terminus of CTNNBL1, as well as from the N-terminus of CDC5L to the C-terminal extended helices of CTNNBL1. Crosslinks also connect K28 at the N-terminus of CDC5L to K185 of CWC15, and K174 in the NLS1 to K92 of CWC15. These results strongly support the notion that CWC15 interacts with the N-terminal region of CTNNBL1, corresponding to the HEAT-like domain. In contrast, the interaction with CDC5L is less certain given the paucity of peptide coverage for some regions, but suggest the involvement of the NLS region of CDC5L and the C-terminal half of CTNNBL1.

**Figure 4.**
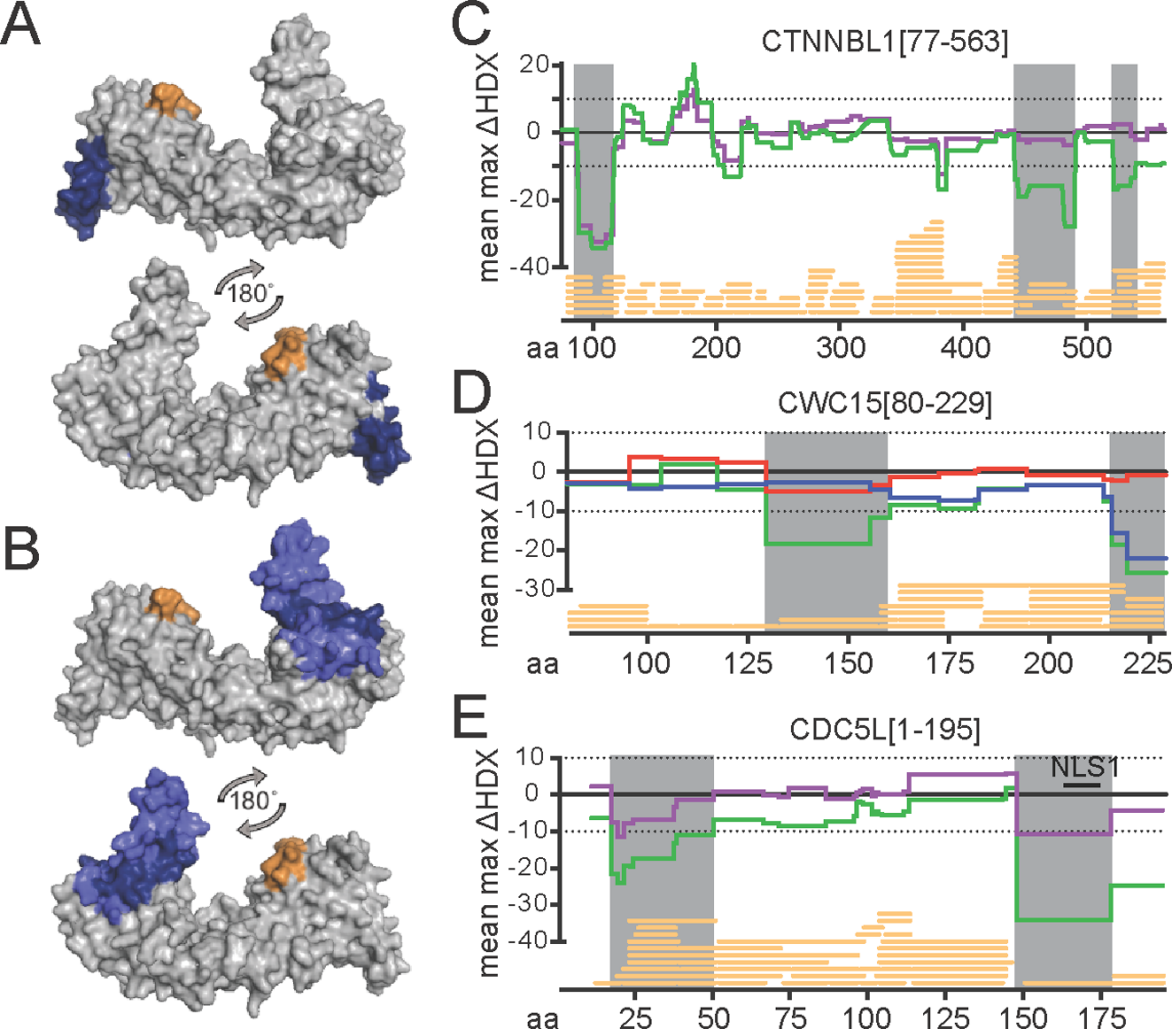
Differential HDX-MS analysis of His-CTNNBL1[77–563], CDC5L[1–195] and CWC15[80–229] complexes. CTNNBL1 surface model displaying areas of interaction with (**A**) CWC15 and (**B**) CDC5L, deduced from above-threshold hydrogen/deuterium content decrease (blue) and increase (orange) (>10%, >0.5D, t-test *P* < 0.05, see Materials and Methods). The view shows the N-terminal region on the left and a 180° horizontal rotation. The mean of maximum exchange (ΔHDX) values plotted for each amino acid corresponding to each protein are shown (see Materials and Methods) in (C) for His-CTNNBL1[77–563] in complex with CWC15[80–229] (purple line); CWC15[80–229] as a dimer with CDC5L[1–195] (red line) or His-CTNNBL1[77–563] (blue line) in D); and for CDC5L[1–195] in association with CWC15[80–229] (purple line) in (E). In all (**C**)–(**E**), the ΔHDX profiles associated with the ternary complex are shown as a green line. Regions with a decrease in exchange above the significance threshold indicated by the dotted lines are shadowed grey. Peptide coverage is indicated by yellow bars at the bottom of each graph on the x-axis which gives the aa numbering. The position of NLS1 in CDC5L is indicated by a black bar.

In order to monitor the dynamics of the interactions in solution and to overcome the limitations of crosslinking data, we used hydrogen-deuterium exchange methods (HDX). The results (see Supplementary Figures S5 and S6 for full listings) are summarized in Figure 4A and B with the differences in deuterium exchange mapped onto the structure of CTNNBL1. In this case we obtained adequate peptide coverage for all three proteins (Figure 4C–E). Comparison of CTNNBL1/CWC15 with free CTNNBL1 reveals strong protection of aa 88–114 of CTNNBL1 (>25% ΔHDX) in the complex, identifying the loop and helices of the first HEAT repeat at the N-terminus of CTNNBL1 as the binding site for CWC15; consistent with the numerous crosslinks observed for this region.

The interaction between CDC5L[1–195] and CTNNBL1 could not be assessed directly under the buffer conditions required for HDX, with low salt precipitating the complex upon binding. We therefore inferred the CDC5L/CTNNBL1 interaction by comparing the differential exchange rates of peptides in the ternary complex and the binary CTNNBL1/CWC15 complex. This strategy identified an extensive area in the C-terminal domain (CTD) of CTNNBL1, including the distal part of the extended coil–coil helices and part of the terminal helices (Figure 4B), which is protected in the ternary complex but not in the CTNNBL1/CWC15 complex. Protection included the groove formed by the two perpendicularly orientated helices aa 522–535, and less but significant protection in two patches aa 246–260 and aa 343–353, on the flank of the solenoid. Binding of CWC15 and CDC5L to CTNNBL1 in the ternary complex also resulted in increased exchange at aa 171–184, possibly indicating either disruption of a CTNNBL1 self-interaction or a small conformational change within the protein when part of the complex.

The results obtained by mapping the HDX exchanges onto the known structure of CTNNBL1 are in agreement with our crosslinking and pull down data thus encouraging us to confidently predict the regions involved in binding for the two proteins where structural information is lacking. The integrated HDX data plotted onto the linear aa sequence of CTNNBL1 (Figure 4C) do clearly identify the N-terminal region as the binding site for CWC15 in the complex of the two (purple line), as well as the protection of the C terminus in the complex that also includes CDC5L (green line). Binding of CTNNBL1 to CWC15 (Figure 4D, blue line) leads to protection of peptides corresponding to the C-terminus of CWC15, aa 214–229. In contrast, no significant difference is found upon CDC5L binding to CWC15 (Figure 4D, red line). This is not too surprising given the high rate of exchange detected for the small CWC15 fragment on its own (Supplementary Figure S5B), consistent with a highly disordered protein. Detecting changes in exchange upon binding of the CDC5L fragment at the threshold chosen (10% ΔHDX) would require a stable and large binding interface between the two proteins. However, a region in the middle of the CWC15 fragment becomes protected within the complex of all three proteins (Figure 4D, green line), likely due to the presence of CDC5L within the complex. In the case of CDC5L, the main area affected by CWC15 binding is the NLS region, aa 150–178 as well as a small region within the Myb domain, aa 20–21 (Figure 4E, purple line), again in full agreement with the mutagenesis and pull down data. Interestingly, the protection observed in CDC5L was more pronounced with all three proteins in complex (Figure 4E, green line), yet the pattern was very similar to that seen for CWC15 binding, and no unique profile for binding of CTNNBL1 could be identified. This might suggest that within the ternary complex, CDC5L and CTNNBL1 do not interact directly but the CDC5L/CWC15 interaction becomes enhanced, alternatively it could indicate an exchange of binding at the NLS region between CWC15 and CTNNBL1. Given the robust interaction between CDC5L and CTNNBL1 seen in the pull down assays mediated by the NLS region, and the large area on the C-terminus of CTNNBL1 that becomes protected by the addition of CDC5L to the complex (Figure 4C), we postulate that the deuterium exchange signature on the NLS region does indeed reflect CTNNBL1 binding. The implication is that the binding site of CWC15 to CDC5L largely overlaps with that of CTNNBL1, an observation predicted by the pull down of CDC5L fragments by both (Figures 1B and 3A). The protection observed in the region encompassing aa20–50 of CDC5L likely reflects binding by CWC15, both in the CDC5L/CWC15 binary complex and in the CTNNBL1/CDC5L/CWC15 ternary complex, as we have no evidence from the pull downs that CTNNBL1 can bind to this region, whereas we did observe a small band in the pull down between CWC15 and the MYB domain of CDC5L (see Figure 3A, fragment D open arrow), suggesting that the MYB domain provides a contact with CWC15.

*CTNNBL1 can self-associate to form a trimer in solution*. Based on crystallization and X-ray structural analysis, it has been suggested that CTNNBL1 by itself forms a homodimer (37). Our crosslinking experiments also suggested a larger species than that predicted for a monomer (Supplementary Figure S3). Indeed, SEC-MALS analyses revealed that both full-length CTNNBL1 and the CTNNBL1[77–563] fragment form complexes with a molecular mass of 186 or 165 kDa respectively (Supplementary Figure S7), corresponding to a homotrimer in solution. The self-association was disrupted both in high salt buffer (0.5 M NaCl), as well as by addition of CWC15[80–229]. We think these results make it unlikely that CTNNBL1 exists as a homodimer or homotrimer *in vivo* for its self-association would be disrupted by CWC15, to form a stable 1:1 pool of CWC15/CTNNBL1, as indeed has been observed by (30).

### CTNNBL1 is required to preserve normal levels of the core Prp19 complex *in vivo*

**Figure 5.**
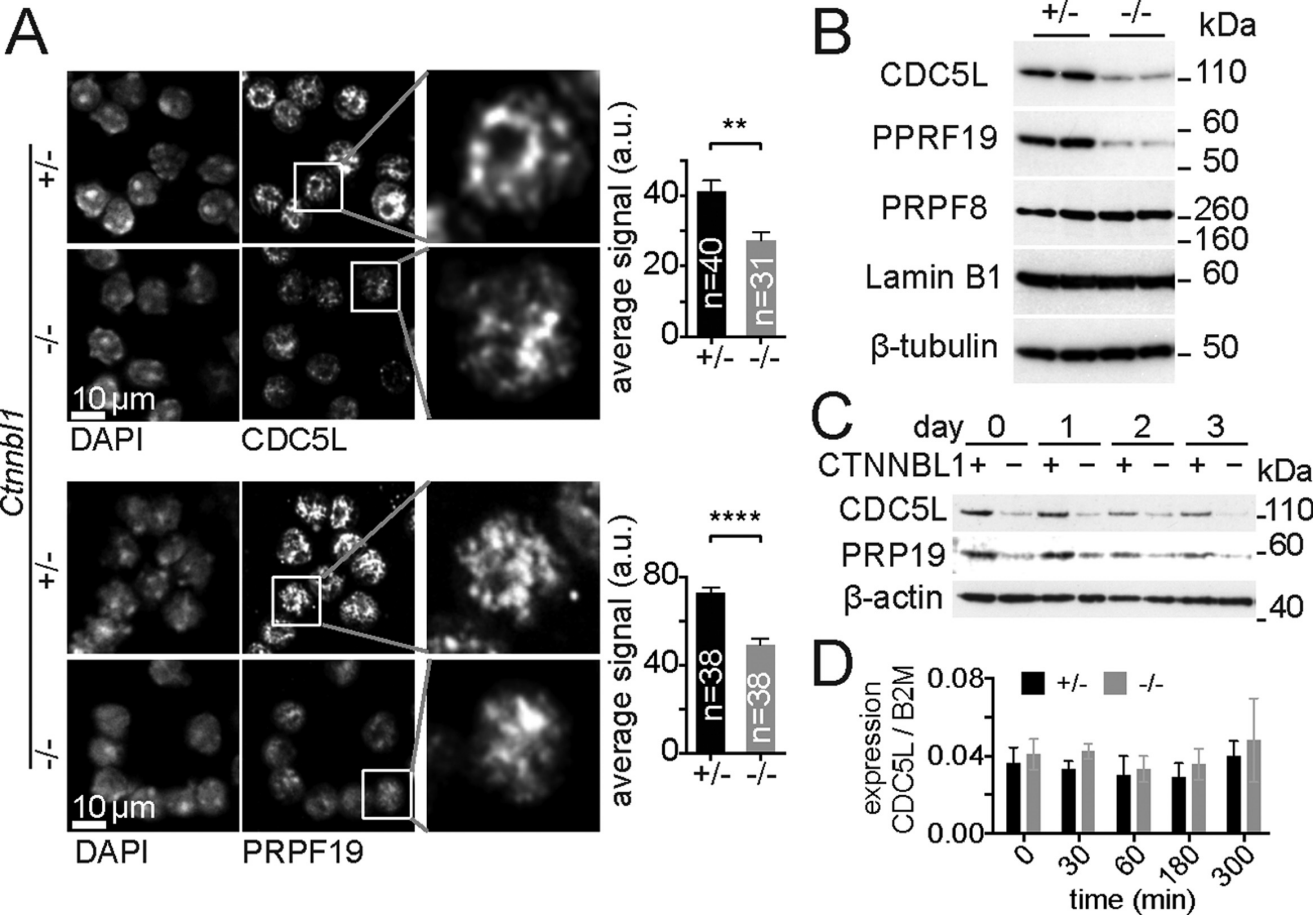
CTNNBL1 deficit impacts the abundance of the CDC5L and PRPF19 in mouse B cells. (**A**) Representative confocal immunofluorescence of methanol fixed mouse resting B cells comparing CDC5L, PRPF19 and DAPI stain in *CD19-Cre Ctnnbl1^+/flx^* controls (+/−) and *CD19-Cre Ctnnbl1^−/flx^* (−/−) mice. The signal in the enlarged box showing a single nucleus has been linearly enhanced in the (−/−) pictures to allow visualization of the characteristic speckled appearance. Histograms show the average ±SEM fluorescence per cell expressed in arbitrary units quantified from the indicated number of cells (*n*) from two separate experiments for each genotype. Asterisks denote the statistical significance of unpaired two-tail t-test, **P = 0.0016 and ****P < 0.0001. (**B**) Abundance of CDC5L and PRPF19 in resting B cells lysates compared to PRPF8, Lamin B1 and β-tubulin from two control (+/−) and two *CD19-Cre Ctnnbl1^−/flx^* (−/−) mice. (**C**) Protein abundance of CDC5L and PRPF19 relative to β-actin shown by western blot in cells lysates derived from proliferating B cells. Control (+) and CTNNBL1 deficient (−) cell cultures were sampled prior to LPS and IL-4 stimulation (day 0) and then at 24 h intervals. (**D**) Relative abundance of CDC5L mRNA relative to β-2 Macroglobulin (B2M) in control (+/−) and CTNNBL1 deficient (−/−) B cells sampled before and 5 h after LPS and IL4 stimulation quantified by q-PCR. The average ratios ± SEM are shown for four biological replicates. Statistical analysis using a two-tail unpaired t-test reported no *P*-values below 0.01.

We have previously shown that CTNNBL1 is required for the viability of mice from mid gestation onwards. While still unexplained, the late embryonic lethality probably reflects the mild phenotype observed in differentiated B cells, with delayed cell growth and cell division (35). Structural similarity to the karyopherins prompted us to consider that the role of CTNNBL1 might depend on its known ability to bind nuclear localization signals. As mentioned before, CTNNBL1 associates with CDC5L in cell lysates (30) and recombinant CTNNBL1 interacts with the NLS region of CDC5L, but not with peptides corresponding to the NLS regions of PRPF19 or PLRG1 (28). We therefore used B cells deficient in CTNNBL1 to determine the intracellular localization of CDC5L. Immunofluorescence on resting splenic B cells from *CD19-Cre Ctnnbl1^−/flx^* mice revealed a 1.5-fold reduction in the amount of nuclear CDC5L signal (Figure 5A and Supplementary Figure S8A). Surprisingly, given that its NLS is not recognized by CTNNBL1, a similar effect was seen for PRPF19, also a core component of the Prp19 complex. The residual nuclear signal displayed the characteristic speckled distribution (11), suggesting that localization was broadly unaffected.

Reduced abundance of both CDC5L and PRPF19 proteins in CTNNBL1 deficient cells was evident in whole cell lysates from resting B cells (Figure 5B), with relative levels of CDC5L reduced between 1.5- and 5-fold (depending on the experiment) and equivalent reductions in PRPF19. In contrast levels of PRPF8 -part of the catalytic core of the spliceosome were unaffected (Figure 5B), indicating that the defect is specific and restricted to the Prp19 core complex, rather than a general defect in splicing components. We observed a similar deficit in the abundance of CDC5L in proliferating B cells (Figure 5C) as well as in asynchronous ES cells cultures (Supplementary Figure S8B), suggesting that the depletion is not restricted to quiescent differentiated cells, but also affects undifferentiated dividing cells. The reduced abundance of CDC5L was not a result of reduced levels of mature messenger RNA, either in resting cells or following activation (Figure 5D). Given that the abundance of PRPF19 which is known not to interact with CTNNBL1 is also affected, whereas the overall localization of either CDC5L or PRPF19 is not, we conclude that the stability of the whole Prp19 complex rather than nuclear import might be dependent on CTNNBL1.

### CTNNBL1 is required for the efficient association of CWC15 and the core CDC5L/Prp19 complex *in vivo*

**Figure 6.**
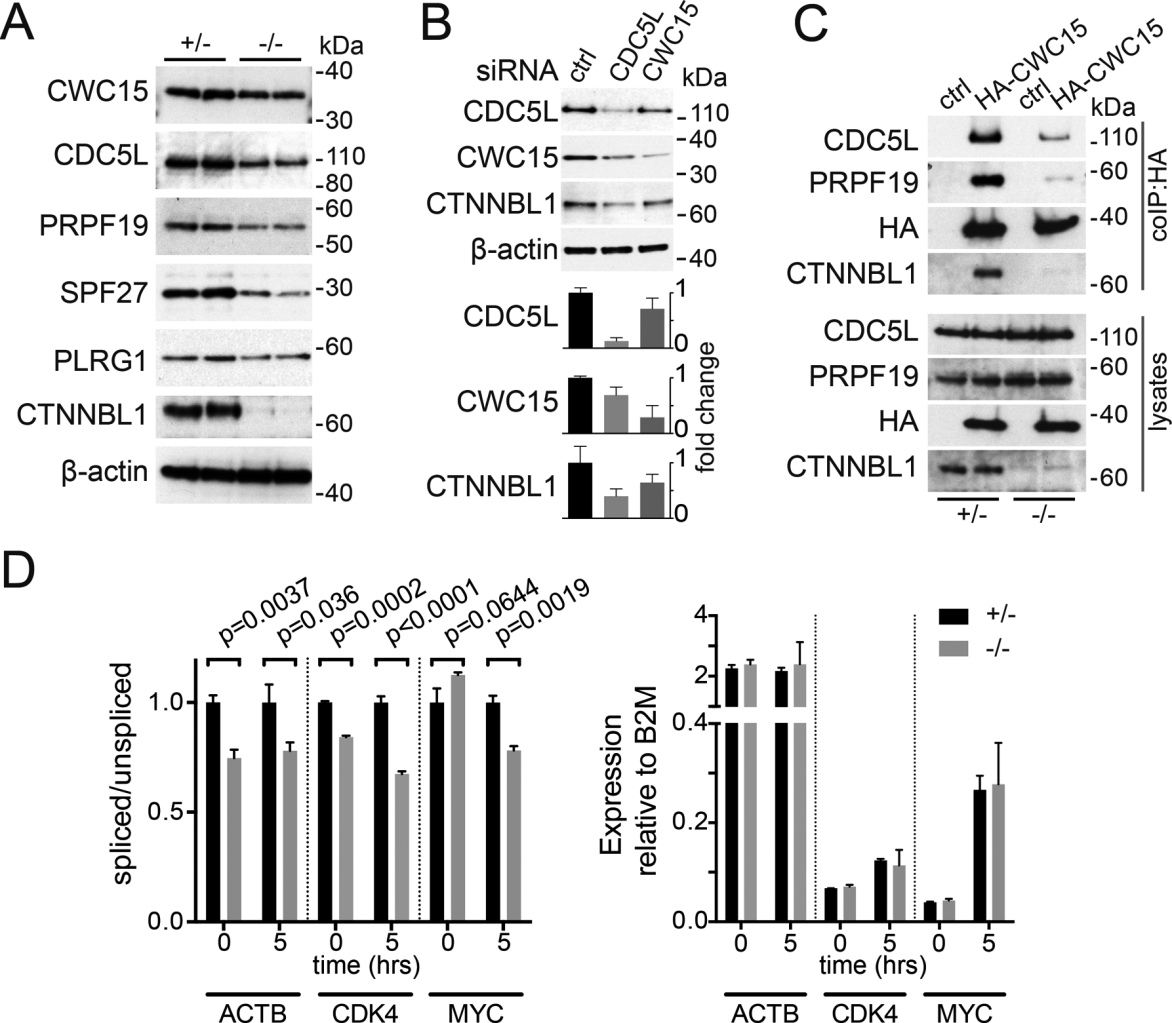
CWC15 depends on CTNNBL1 for efficient association with the Prp19 complex *in vivo*. (**A**) Abundance of the endogenous Prp19 complex proteins relative to β-actin in resting B cells from two control mice (+/−) and two *CD19-Cre Ctnnbl1^−/flx^* (−/−) mice. (**B**) Abundance of CDC5L, CWC15 and CTNNBL1 in cell lysates of human U2OS cells 24 h after transduction with siRNA pools specific for either CDC5L or CWC15. Non-targeting siRNA pools were used as control. A representative western is shown, with abundance of the proteins relative to β-actin quantified below showing the average ±SEM from three independent experiments. (**C**) Immunoprecipitation (IP) of mouse CDC5L and PRPF19 in retrovirally transduced B cells expressing human HA-CWC15 or empty vector (ctrl). Transduced B cells from either *CD19-Cre Ctnnbl1^+/flx^* control mice (+/−) or *CD19-Cre Ctnnbl1^−/flx^* (−/−) were stimulated with LPS and IL-4 for 4 days prior to lysis and purification on anti-HA resin. Lysates show 1.5% of the input used for IP. The amount of lysate used in the IPs was adjusted to account for the reduced levels of CDC5L and PRPF19 in the (−/−) samples. Size markers (kDa) are indicated. (**D**) Splicing efficiency estimated from the ratio of first intron inclusion versus spliced messenger RNA measured by q-PCR on total RNA extracted from resting or activated B cells from either *CD19-Cre Ctnnbl1^+/flx^* control (+/−) or *CD19-Cre Ctnnbl1^−/flx^* (−/−) mice at the indicated times post activation. The average ±SEM ratios are obtained from three independent mice. Statistical *P* values of an unpaired one-tail t-test for each time point are shown. The right histograms show the relative abundance mRNA relative to β-2 Macroglobulin (B2M) in control (+/−) and CTNNBL1 deficient (−/−) B cells. Statistical analysis using a two-tail unpaired t-test reported no *P*-values below 0.1.

*In vivo*, CTNNBL1 appears to be required to maintain normal levels of CDC5L and PRPF19. A dependence on other components within the Prp19 complex has been previously observed (15,17) with abundance of one core protein affected by the levels of another. We therefore confirmed that levels of all core proteins are indeed disrupted in CTNNBL1 deficient B cells (Figure 6A). We then tested whether depletion of CWC15 itself was also sufficient to affect the levels of the proteins associated within CTNNBL1. Indeed siRNA-mediated depletion of human CWC15 in U2OS cells does disrupt normal levels of CDC5L protein as well as CTNNBL1 (Figure 6B), with the reciprocal effect also elicited by depletion of CDC5L. This confirms the interdependence of the three proteins in the CTNNBL1 sub-complex for their stability *in vivo*, and functionally links the stability of the whole Prp19 complex to the presence or absence of CTNNBL1 and CWC15.

Our *in vitro* dissection of the architecture of the CWC15/CTNNBL1/CDC5L complex suggested that the interaction between CDC5L and CWC15 might be rather weak in the absence of CTNNBL1. We therefore wondered whether the differences in binding might be reflecting the requirement for CTNNBL1 to stabilize the association of CWC15 with the rest of the complex *in vivo*. Overexpression of a HA tagged CWC15 allowed for the efficient immunoprecipitation of CDC5L (and PRPF19), as well as CTNNBL1, however these interactions were diminished in B cells deficient for CTNNBL1 (Figure 6C). The abundance of the endogenous CWC15 appeared largely unaffected or only marginally reduced in the absence of CTNNBL1 (Figure 6A), which is compatible with the notion that the stability of CWC15 itself is not greatly compromized, unless in the context of the rest of the core Prp19 complex. The defect in Prp19 complex levels results in a subtle reduction in the efficiency of splicing, evident as an increase in intronic retention observed in the first intron of several RNAs tested (Figure 6D). The deficit is also detected early post activation, presumably reflecting the burden imposed by processing newly synthesized transcripts. The overall levels of mature mRNA are not overtly affected (as was the case for CDC5L, Figure 5D and previously documented (35)), suggesting that the partial depletion of the Prp19 complex elicited by CTNNBL1 deficiency might only become limiting when transcription demands are increased or for particular transcripts.

## DISCUSSION

CTNNBL1 has consistently been found associated with the Prp19 complex, yet was not considered to be part of the core, as it dissociates from the CDC5L-SPF27-PRPF19-PLRG1 complex under high salt conditions and upon heparin treatment (1,30). Moreover, CTNNBL1 is not essential for cell viability in contrast to other members of the Prp19 complex (39). Nevertheless, our results demonstrate that CTNNBL1 is functionally interacting with the complex by maintaining the cellular levels of core complex proteins. A reduction in the stability of the Prp19 complex has previously been documented; depletion of components of the core, such as SPF27 by siRNAs resulted in a reduction in the abundance of the rest of the complex (17). Similarly, depletion of PRPF19 led to reduced levels of CDC5L and vice versa (15,16), suggesting this may be a common phenotype for Prp19 core proteins. Our results highlight the dependence of the core complex for its stability on the assembly of one of its essential components, CWC15, which in turn requires CTNNBL1 for efficient association with the rest of the core via CDC5L. Our results also reinforce the robustness of the HDX-MS method to provide information for even weak interactions of proteins in solution when other structural or biochemical methods might not be suitable. The functional importance of the regions identified by HDX is supported by sequence conservation across species. The highly conserved CTD of CTNNBL1 mediates interaction with a highly disordered region (Supplementary Figure S9) surrounding NLS1 of CDC5L, which is also highly conserved between the human and yeast proteins (>90% identical in *S. pombe* Cdc5, *S. cerevisiae* CEF1 and human CDC5L). By comparison, the C-terminal spliceosome interacting region of CDC5L is overall less conserved between yeast and humans as is the conservation between human and *Sc*. CWC15. Interestingly, the C-terminal region of CWC15 that we identify as binding CTNNBL1 is also conserved across the three species, although less so in *S. cerevisiae* (which lacks CTNNBL1). Although the *S. pombe* homologue of CTNNBL1 (SPAC1952.06c) has not been detected in spliceosomal/NTC complexes (yeast homologous Prp19 complex) (5,25), it is likely that the interactions with *S. pombe* Cwf15 or Cdc5 (the homologues of human CWC15 and CDC5L respectively) are conserved.

**Figure 7.**
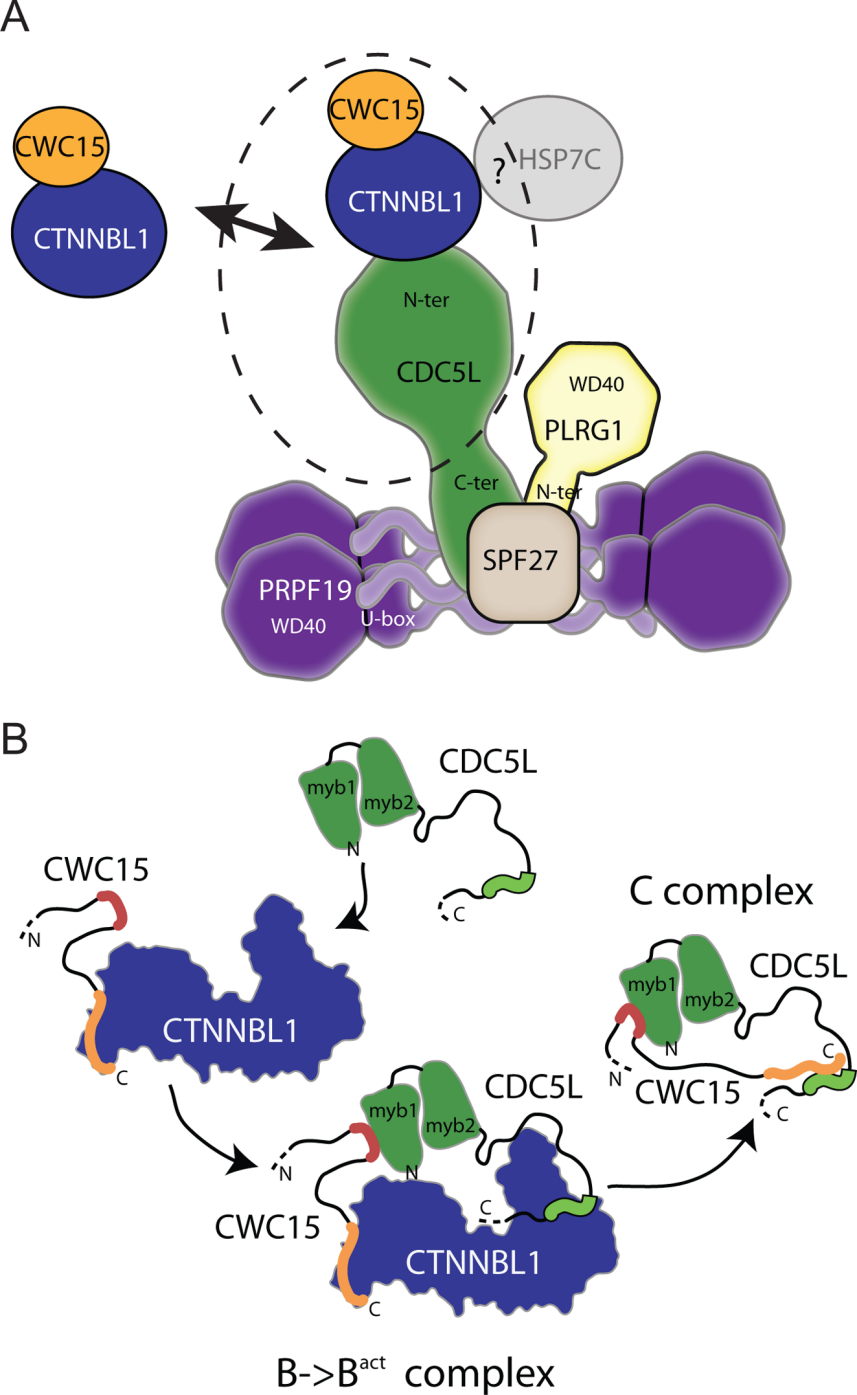
Architecture of the CWC15, CTNNBL1 and CDC5L sub-complex in relation to the core Prp19 complex. (**A**) Architecture of the mammalian Prp19 complex. Based on biochemical and structural information, including negative staining electron microscopy of purified proteins, the core Prp19 complex is thought to consist of an extended structure formed by a tetramer of PRPF19 with the coiled-coil regions in its centre and the WD40 domains on the outside. A single copy of CDC5L is associated with the coiled-coil region of the PRPF19 tetramer through its less conserved carboxy-terminal region, designated as the spliceosomal interacting domain, whereas the more conserved N-terminal portion containing the MYB domains and NLS region is thought to associate with CTNNBL1. One copy of SPF27 is also found in association with the central portion of the PRPF19 tetramer which also contains the N-terminal region of PLRG1. CWC15 and CTNNBL1 have been described as a separate stable complex outside the Prp19 core, but are also found in association with the Prp19 complex through interaction with the N-terminal region of CDC5L. The precise configuration of the CTNNBL1/CWC15/CDC5L sub-complex (dotted ellipse) within the core Prp19 complex had not been fully defined but was expected to adopt a linear conformation. The positioning of HSP7C, a sub stoichiometric component has not been characterized (Model based on Grote *et al*. (30)). (**B**) Changes in the interactions between CWC15, CTNNBL1 and CDC5L in relation to the different spliceosomal complexes. CTNNBL1 and CWC15 form a stable dimer with the N-terminal of CTNNBL1 bound to the C-terminus of CWC15 (orange). In the B and B^act^ forms of the spliceosome, the Prp19 complex is stabilized by the presence of CTNNBL1, enhancing the otherwise weak interactions between CWC15 and CDC5L. CTNNBL1 acts as bridge, binding the N-terminal NLS region of CDC5L (green squiggle) through its CTD region and the C-terminus of CWC15 through its N-terminal HEAT-like region. This allows for a three-way interaction facilitating binding between the MYB domain of CDC5L (green triangular forms) and a region in the central portion of CWC15 (red squiggle). This stable three-way interaction maintains the normal levels of the Prp19 complex in B and B^act^ spliceosomes. At the later C stage of the spliceosome complex, CTNNBL1 is not present, and the NLS region of CDC5L can interact with the C terminal region of CWC15.

Integrating the pull down and HDX-MS data of the CTNNBL1/CWC15/CDC5L sub-complexes, the simpler interpretation would be to place CTNNBL1 at the centre of a linear complex between CWC15 bound at its N-terminal portion and CDC5L bound to the CTD, a configuration in agreement with that proposed by Grote *et al*. (30) (Figure 7A). This configuration however would keep CWC15 and CDC5L quite far apart potentially preventing the interaction between the two within the complex. This poses the question of how both proteins maintain the interaction at the later stages, post activation of the catalytic stage of the spliceosome, when CTNNBL1 is no longer found in the complex. Interestingly, our data reveal that the binding site of CTNNBL1 on CDC5L, i.e. the NLS region, largely overlaps with that of CWC15, so that CTNNBL1 and CWC15 may be competing for binding to CDC5L. However, the binding is not exactly the same for the two proteins, with the MYB domain of CDC5L contributing to the interaction with CWC15 and the NLS3 peptide unable to suffice for binding to CWC15, as is for CTNNBL1. Here we have limited our analysis to the binding of the N-terminal section of CDC5L, i.e. [aa1–195], as we found that to be the minimal domain required for interaction. It is clear that additional regions of the N-terminal domain of CDC5L, such as the NLS3 region, do make contact with CTNNBL1, and that the interaction surface may be more extensive. Ahn *et al*. have recently described a fragment of CDC5L, including aa141–377, that binds to the CTD region of CTNNBL1 (37). The binding is dependent on residues E264, D308 and D315, as well as a region including E178-E179 towards the N-terminus of CTNNBL1. These results are compatible with our findings and would suggest that full-length CDC5L might extend towards the N-terminus of CTNNBL1, where it could make contact with CWC15.

Our biochemical results would suggest that a self-interaction of CTNNBL1 (either a homotrimer or as suggested by (37) a homodimer) is readily displaced by CWC15, and that this complex exists as a stable 1:1 pool of CWC15/CTNNBL1 as described *in vivo* by (30), whereas the interaction between CWC15 and CDC5L is rather weak. Nonetheless, efficient association of CWC15 and CDC5L—at least *in vitro*—does seem to rely on the presence of CTNNBL1, acting as a bridge between the two proteins. And as we show here, *in vivo* overexpressed CWC15 is again impaired in its ability to bind full-length CDC5L and the rest of the Prp19 complex in the absence of CTNNBL1. Taken altogether, our data support the stoichiometry of CDC5L in the Prp19 complex *in vivo* as reported by Schmidt *et al*., with a single molecule of CDC5L associated with one copy of CTNNBL1 and one copy of CWC15 (31),

Extensive biochemical and functional data have demonstrated that the association of the Prp19 complex with the spliceosome prior to catalytic activation (3,26,40) is a requirement for splicing to proceed (1). Most of the proteins in the Prp19 core complex including CWC15 remain in the activated spliceosome until splicing is completed, whereas CTNNBL1 is part of the complex when it is in the B^act^ state, but dissociates during the B* to C transition (3,26,41). The presence of CTNNBL1 in the spliceosomal B^act^ complex suggests that the association might be important for the overall function of the Prp19 complex, rather than just aiding its assembly. The small defect in splicing efficiency in *Ctnnbl1* knockout cells is compatible with the CTNNBL1-containing Prp19 complex's reported role in stabilizing the step 1 conformation of the Prp19/snRNP (1,42), with the stability of the Prp19 complex itself dependent on the efficient association of CWC15 to CDC5L. In this scenario, CWC15 would interact with the Prp19 complex in a different manner in the C complex, presumably through direct binding to CDC5L. Furthermore, our data is compatible with the notion that CWC15 and CTNNBL1 might exchange places in the C complex, given that their binding site on CDC5L is largely overlapping (Figure 7B). CDC5L undergoes phosphorylation changes at different stages of the spliceosome which may stabilize the interaction between CWC15 and CDC5L in the C complex; alternatively, the stabilising function of CTNNBL1 might be replaced by the addition of HSP7C, whose abundance increases in complex C (26).

Deficit in CTNNBL1, despite impairing mammalian development, has a minor effect in cells, and as we show here diminishes the abundance of core Prp19 components, but not sufficiently to lead to a major global defect in splicing which would be expected to abrogate cell autonomous functions like proliferation or differentiation. Nonetheless, in addition to the previously reported delay in cell cycle entry (35), we detect a subtle effect on splicing efficiency in CTNNBL1 deficient cells, in particular at the stage when RNA production is increased in the early transition from quiescent B cells to rapidly proliferating blasts. However, this deficit is not apparent later on (35), suggesting the reduced levels of the Prp19 complex are not limiting once the cells are actively dividing. siRNA-mediated inhibition of CDC5L and CWC15 did not affect the viability of U2OS cells (data not shown), suggesting that even with a significant reduction in Prp19 complex proteins, splicing may only be mildly affected. It is possible that a subtle splicing deficit becomes critical for certain genes at certain stages of development or under stress situations, with cumulative defects (43,44), leading to lethality. Alternatively, reduced stability of the Prp19 complex might result in reduced abundance of PRPF19 limiting other of its cellular functions outside the spliceosome which impact on the proliferative capacity of the cells (45) eventually leading to developmental failure.

## Supplementary Material

SUPPLEMENTARY DATA
