# Supplementary material for: CTNNBL1 facilitates the association of CWC15 with CDC5L and is required to maintain the abundance of the Prp19 spliceosomal complex
Source: Nucleic Acids Res. 2015 Jun 29;43(14):7058–69. doi: 10.1093/nar/gkv643 (PMC4538830; doi:10.1093/nar/gkv643)
Supplement: SUPPLEMENTARY DATA [file supp_43_14_7058__index.html]

CTNNBL1 facilitates the association of CWC15 with CDC5L and is required to maintain the abundance of the Prp19 spliceosomal complex — SUPPLEMENTARY DATA 

# CTNNBL1 facilitates the association of CWC15 with CDC5L and is required to maintain the abundance of the Prp19 spliceosomal complex

## SUPPLEMENTARY DATA

- SUPPLEMENTARY DATA
