# Supplementary material for: CTNNBL1 facilitates the association of CWC15 with CDC5L and is required to maintain the abundance of the Prp19 spliceosomal complex
Source: Nucleic Acids Res. 2015 Jun 29;43(14):7058–69. doi: 10.1093/nar/gkv643 (PMC4538830; doi:10.1093/nar/gkv643)
Supplement: SUPPLEMENTARY DATA [file supp_gkv643_nar-01372-a-2015-File009.pdf]

CTNNBL1 facilitates the association of CWC15 with CDC5L and is required to maintain the abundance of the Prp19 spliceosomal complex

Febe van Maldegem[1*$], Sarah Maslen[1], Christopher M Johnson[1], Anita Chandra[1£] Karuna Ganesh[2], Mark Skehel[1] and Cristina Rada[1*]

SUPPLEMENTARY DATA

Supplementary Methods

Expression vectors. Plasmids with CTNNBL1 and its mutants have been described previously (1, 2). Full length and truncated versions of CWC15 and CDC5L were PCR amplified and cloned using the primers and enzymes listed in Table S1. pOPTM was a kind gift of Dr. O. Perisic, Cambridge.

Protein crosslinking. After purification,10 mM of each protein was mixed in crosslinking buffer (see buffer list), crosslinking was initiated by addition of a mix of BS3-d0 and BS3-d4 (Bis(sulfosuccinimidyl) suberate deuterated and non-deuterated crosslinking reagent mix, BS3-d4/d0 -with a spacer arm of 11.4 Å) up to 0.5 mM, and terminated with a final concentration of 50 mM Tris pH7.5 after 0, 15 and 30 min. Complexes were separated by SDS-PAGE and gel bands were cut out and digested with trypsin. The resulting peptides were analysed by nano-scale capillary LC-MS/MS using an Ultimate U3000 UPLC (ThermoScientific Dionex) to deliver a flow of approximately 300 nL/min. A C18 Acclaim PepMap100 5 µm, 100 µm x 20 mm nanoViper trapped the peptides prior to separation on a C18 Acclaim PepMap100 3 µm, 75 µm x 250 mm nanoViper (both from ThermoScientific Dionex). Peptides were eluted with a gradient of acetonitrile. The analytical column outlet was directly interfaced with a LTQ-Velos Orbitrap mass spectrometer (ThermoScientific) operated in DDA (Data Dependent Acquisition) mode, where an initial MS scan is followed by fragmentation of the top 10 ions. The resulting data were analysed using Hekate cross-linking software (3) and all potential cross-links were manually validated.

Hydrogen deuterium exchange mass spectrometry (HDX-MS). Proteins were dialysed against exchange buffer but with MPW instead of D_2O (see buffer table) and then diluted as single proteins or combined ([CWC15 + CDC5L], [CWC15 + CTNNBL1] or [CWC15 + CDC5L + CTNNBL1]) to 1 mg/ml for His-CTNNBL1 and 0.5 mg/ml for CWC15 and CDC5L in a volume of 10 µl. The protein solutions were mixed with 40 µl of exchange buffer (with D_2O) for 3 seconds on ice and 3, 30, 300 and 3000 seconds at room temperature (22°C) before quenching by the addition of 20 µl ice cold quench buffer (buffer list) and snap freezing in liquid nitrogen. Each time point and protein combination was done in triplicate.

The quenched protein samples were rapidly thawed and subjected to proteolytic cleavage by pepsin followed by reversed phase HPLC separation essentially as previously described (4). The

protein was passed through an immobilized pepsin column, 2.1 x 30 mm (Porozyme, ABI) at 230 µl/min for 3 min and the peptic peptides trapped and desalted on a 2.1 x 5 mm C18 trap column (Acuity BEH C18 Van-guard pre-column, 1.7 µm, Waters). Trapped peptides were subsequently eluted over 12 min using a 5-36% gradient of acetonitrile in 0.1%v/v formic acid at 40 µl/min. Peptides were separated on a reverse phase column (Aquity UPLC BEH C18 column 1.7 µm, 100 mm x 1 mm (Waters). Peptides were detected on a Xevo G2 Q-TOF (Waters) acquiring over a mass range from 300 to 1400 m/z, with the standard electrospray ionization (ESI) source and lock mass calibration using [Glu1]-fibrino peptide B (500 fmol/µl). The mass spectrometer was operated at a source temperature of 80°C, and a spray voltage of 2.6 kV. Spectra were collected in positive ion mode.

Peptide identification was performed by data dependent acquisition using a 3-35% gradient of acetonitrile in 0.1%v/v formic acid over 120 min with a Xevo G2 Q-TOF (Waters). This was supplemented with a shorter 20 min gradient separation to identify and correct the retention time for all peptide samples. The resulting MS/MS data was analysed using the Mascot database search engine (Matrix Science) using an MS tolerance of 5 ppm and a MS/MS tolerance of 0.15 Da. Mass analysis of the peptide centroids was performed as described previously, using the software HD-Examiner (Sierra Analytics) (4). Only peptides with a Mascot score >15 were considered. The first round of analysis and identification were performed automatically by the HD-Examiner software, however, all peptides (deuterated and non-deuterated) were manually verified at every state and time point for the correct charge state, m/z range, presence of overlapping peptides, and the expected retention time. Deuterium incorporation was not corrected for back-exchange and represents relative, rather than absolute changes in deuterium levels. Changes in H/D amide exchange in any peptide may be due to a single amide or a number of amides within that peptide. All time points in this study were prepared at the same time. Individual time points were acquired on the mass spectrometer on the same day.

Buffer list

Triton lysis buffer

20 mM Tris-Cl pH8, 125 mM NaCl, 1 mM $MgCl_2$, 10% glycerol

Bacterial protein expression and purification buffers

Lysis Buffer: As wash buffer, but supplemented with: Lysozyme, 1 mg/ml (Sigma Aldrich), EDTA-free protease inhibitors (Roche) and Benzonase, 100 U/ml (Merck Millipore)
Wash Buffer for CTNNBL1: 50 mM Na_2HPO_4/NaH_2PO_4 pH7.4, 500 mM NaCl, 1 mM $MgCl_2$. Wash Buffer for CDC5L and CWC15: 50 mM Tris pH7.4, 150 mM NaCl, 5mM $MgCl_2$, 5 mM 2-Mercaptoethanol, 0.1% Triton X-100.

Ion exchange buffers:

For CTNNBL1: (Buffer A) 20 mM Tris pH8.0, 50 mM NaCl, 5 mM 2-Mercaptoethanol and (Buffer B) As buffer (A) but 1M NaCl
For CDC5L: (Buffer A) 50 mM Tris pH7.0, 150 mM NaCl, 5mM $MgCl_2$, 5 mM 2-Mercaptoethanol and (Buffer B) As buffer (A) but 1M NaCl
For CWC15: (Buffer A) 50 mM Tris pH8.0, 150 mM NaCl, 5mM $MgCl_2$, 5 mM 2-Mercaptoethanol and (Buffer B) As buffer A, but 1M NaCl

Pull down buffer

20 mM Hepes pH8.0, 110 mM KAc, 2 mM MgAc, 20 mM Imidazole, 10% glycerol, 0.2% Tween 20, 5 mM 2-Mercaptoethanol

Crosslinking buffer/SEC-MALS buffer

20 mM Hepes pH8.0, 110 mM KAc, 1 mM DTT

HDX - Exchange buffer

10 mM Hepes pH8.0, 50 mM NaCl, 1 mM DTT, in 99% Deuterium

3.5x HDX - Quench buffer

2 M Guanidinium HCl, 2.6% formic acid in Millipore deionised water

REFERENCES

1. Ganesh, K., Adam, S., Taylor, B., Simpson, P., Rada, C. and Neuberger, M. (2011) CTNNBL1 is a novel nuclear localization sequence-binding protein that recognizes RNA-splicing factors CDC5L and Prp31. *J. Biol. Chem.*, **286**, 17091–17102.

2. Ganesh, K., van Maldegem, F., Telerman, S.B., Simpson, P., Johnson, C.M., Williams, R.L., Neuberger, M.S. and Rada, C. (2014) Structural and mutational analysis reveals that CTNNBL1 binds NLSs in a manner distinct from that of its closest armadillo-relative, karyopherin α. *FEBS Letters*, **588**, 21–27.

3. Holding, A.N., Lamers, M.H., Stephens, E. and Skehel, J.M. (2013) Hekate: software suite for the mass spectrometric analysis and three-dimensional visualization of cross-linked protein samples. *J. Proteome Res.*, **12**, 5923–5933.

4. Burke, J.E., Vadas, O., Berndt, A., Finegan, T., Perisic, O. and Williams, R.L. (2011) Dynamics of the phosphoinositide 3-kinase p110δ interaction with p85α and membranes reveals aspects of regulation distinct from p110α. *Structure*, **19**, 1127–1137.

5. Peng, K., Radivojac, P., Vucetic, S., Dunker, A.K. and Obradovic, Z. (2006) Length-dependent prediction of protein intrinsic disorder. *BMC Bioinformatics*, **7**, 208.

6. Obradovic, Z., Peng, K., Vucetic, S., Radivojac, P. and Dunker, A.K. (2005) Exploiting heterogeneous sequence properties improves prediction of protein disorder. *Proteins*, **61 Suppl 7**, 176–182.

7. Romero, P., Obradovic, Z., Li, X., Garner, E.C., Brown, C.J. and Dunker, A.K. (2001) Sequence complexity of disordered protein. *Proteins*, **42**, 38–48.

Supplementary Tables

Table S1.

List of plasmids and primers used.

Primer	Construct name/Primer sequence	Vector	Enzyme
	HA-CWC15<br>GST-CWC15	pcDNA3.1-3HA<br>pGEX-6P-1	
CWC15[1-229]_fw<br><br>CWC15[1-229]_rev	ATAT-GGATCC-ATGACAACAGCAGCCAGGC<br><br>ATAT-GAATTC-<br>CTATTTAATATATTTCTCCATGAACTTTTTGTG		BamHI<br><br>EcoRI
	HA-CWC15[1-79]<br>GST-CWC15[1-79]	pcDNA3.1-3HA<br>pGEX-6P-1	
CWC15[1-229]_fw<br><br>CWC15[1-79]_rev	ATAT-GAATTC-<br>CTATTTAATATATTTCTCCATGAACTTTTTGTG<br><br>ATAT-GAATTC-<br>CTATGGACGATCCCTATTTTTCTCTC		BamHI<br><br>EcoRI
	HA-CWC15[80-229]<br>GST-CWC15[80-229]	pcDNA3.1-3HA<br>pGEX-6P-1	
CWC15[80-229]_fw<br><br>CWC15[1-229]_rev	ATAT-GGATCC-ACCCGAGAACATACAACCTCC<br><br>ATAT-GAATTC-<br>CTATTTAATATATTTCTCCATGAACTTTTTGTG		BamHI<br><br>EcoRI
	MBP-CDC5L[1-500]	pOPTM	
CDC5L[1-500]_fw<br><br>CDC5L[1-500]_rev	AAT-CATATG-CCTCGAATTATGATCAAG<br><br>AAT-ACGCGT-TCAGAATTTTGACTTTAAAGTCTC		NdeI<br><br>MluI
	MBP-CDC5L[1-271]	pOPTM	
CDC5L[1-500]_fw<br><br>CDC5L[1-271]_rev	AAT-CATATG-CCTCGAATTATGATCAAG<br><br>AAT-ACGCGT-<br>TCAGAATAGCTGATGGTAAATCAG		NdeI<br><br>MluI
	MBP-CDC5L[1-195]	pOPTM	
CDC5L[1-500]_fw<br><br>CDC5L[1-195]_rev	AAT-CATATG-CCTCGAATTATGATCAAG<br><br>AAT-ACGCGT-TCAGCCAGCTGCTCGAAGTTCTC		NdeI<br><br>MluI
	MBP-CDC5L[1-165]	pOPTM	
CDC5L[1-500]_fw	AAT-CATATG-CCTCGAATTATGATCAAG		NdeI

CDC5L[1-165]_rev	AAT-ACGCGT-<br>TCACTTCTTTCCCTGAGTATTAGCC		MluI

Table S2.

List of primers used for quantitative PCR.

Primer	Primer sequence	
CDC5L<br>_fw<br>_rev	 CCAGTGCTTGGAACACTATGAG<br>CATCCATGTCTATCGGGTCA	 exon 3<br>exon 5
B2M<br>_fw<br>_rev	 GTCGCGGTCGCTTCAGTCGT<br>TCCGGTGGGTGGCGTGAGTA	 exon 1<br>exon 2
ACTB<br>_fw<br>_rev1<br>_rev2	 CTGTCGAGTCGCGTCCACCC<br>ACATGCCGGAGCCGTTGTCG<br>CACCCGCCCATGGTGTCCG	 exon 1<br>exon 2<br>intron 1-2
CDK4<br>_fw<br>_rev1<br>_rev2	 CACCTCCAGAGCTCTTAGCC<br>GATCTCGGGCTTTGTACACC<br>TCCAAAATCACACCCTTGC	 exon 1<br>exon 2<br>intron 1-2
MYC<br>_fw<br>_rev1<br>_rev2	 CTCCTCGAGCTGTTTGAAGG<br>AATAGGGCTGTACGGAGTCG<br>TGTCAAAAGCACAGGAATGG	 exon 1<br>exon 2<br>intron 1-2

Table S3

Species nomenclature for components of the mammalian Prp19 complex and spliceosome proteins referred to in the text. The known protein homologues in yeast are included for reference.

	Homo Sapiens	*Mus Musculus*	*Schizosaccharomyces pombe*	*Saccharomyces cerevisiae*
CTNNBL1	CTNNBL1	CTNNBL1[EL]	SPAC1952.06c[NE]	not present
CWC15	CWC15/AD002	CWC15	Cwf15/<br>SPBC337.06c*	CWC15/<br>YDR163W[NE]
CDC5L	CDC5L	CDC5L	Cdc5/<br>SPAC644.12*	CEF1/NTC85/<br>YMR213W*
PRPF19	PRPF19/PSO4/<br>SNEV	PRPF19[EL]	Prp19/Cwf8/<br>SPAC29A4.08c*	PRP19/PSO4/<br>YLL036C*
PLRG1	PLRG1/PLR1	PLRG1[EL]	Prp5/Cwf1/	PRP46/NTC50

			SPBP22H7.07*	YPL151C*
SPF27	SPF27/BCAS2/ DAM1	SPF27/BCAS2	Cwf7/Spf27/ SPBC28F2.04c*	SNT309/NTC25/ YPR101W[NE]
PRPF8	PRPF8/RP13/p2 20	PRPF8/DBF3	Spp42/Cwf6/ SPAC4F8.12c*	PRP8/USA2/ YHR165C*
HSP7C	HSP7C/HSC70/ HSPA8/HSP73	HSP7C/HSPA8	multiple homologues	multiple homologues

Official nomenclature is indicated first. * Known essential and [NE] known non-essential proteins. [EL] indicates reported mouse embryonic lethality

Supplementary Figure Legends

Figure S1. Mutants of CTNNBL1 retain their interaction with N-terminal portion of CDC5L.

CDC5L[1-195] MBP fusion purified on amylose resin in the presence of wild type or mutant His-CTNNBL1[77-563] (as described in Ganesh, et al. 2014) separated by SDS-PAGE. WT, CTNNBL1[77-563]; mutant 1 [77-520], mutant 2 [D153A, N154A, T155A, D156A], mutant 3 [L321A, M322A, H359A, I362A], mutant 4 [D170A, D172A], mutant 5 [H216A, N217A], mutant 6 [Q166A], mutant 7 [E223A, N224A, Y261A], mutant 8 [E264A, I268A], mutant 9 [D315A, S319A, H359A], WT, GST control. Asterisks indicate bait of pull downs. Input proteins (lower panel) and size markers (kDa) are shown.

Figure S2. The NLS3 peptide of CDC5L is not sufficient to bind CWC15.

Pull down of recombinant human CWC15[80-229] (lanes 1 and 3) or His-CTNNBL1[77-563] (lanes 2 and 4) by GST-NLS3 (lane 1-2) or GST only as a control (lane 3-4) on glutathione sepharose 4B beads. Asterisks indicate bait of the pull down assays. Input proteins (lower panel) and size markers (kDa) are indicated.

Figure S3. Inter-protein crosslinks of CTNNBL1, CDC5L and CWC15 complexes in solution. Mobility of protein complexes separated by SDS-PAGE after crosslinking with Bis(sulfosuccinimidyl) suberate deuterated and non-deuterated crosslinking reagent mix (BS3-d4/d0) for the indicated times. The human recombinant proteins used as input are indicated on the top, while the resulting complexes are denoted on the right of each panel. Size markers (kDa) are indicated. Cross-links for: A) each protein on its own; B) a 1 to 1 molar mix of two of the proteins at a time and C) an equimolar mix of the three proteins. A faint band corresponding to the molecular weight of a dimer of the CDC5L fragment is indicated in panel A.

Figure S4. Chemical crosslinking identifies interaction sites between CTNNBL1, CDC5L and CWC15.

Graphical representation of the crosslinks found between A) recombinant human CTNNBL1 and CWC15, B) CTNNBL1 and CDC5L, and C) CWC15 and CDC5L. Crosslinks between peptides within the same protein are visualised with grey lines. Increased line width denotes the frequency in which the crosslink was detected. A surface model of CTNNBL1 is shown in gray with surface lysines coloured in yellow. Linear sequences of the CWC15[80-229] and CDC5L[1-195] fragments analysed are shown, with the aa numbering and the position of lysines indicated. Secondary structure predictions are shown, with helices and beta sheets marked with H and S in red and yellow blocks respectively. D) Coverage of the un-crosslinked peptides for each protein, with the frequency (y-axis) of each amino acid along the protein (aa) detected in the recovered peptides.

Figure. S5. Global hydrogen deuterium exchange levels over time of the different proteins and complexes in solution.

Exchange levels for peptides from A) His-CTNNBL1[77-563], B) CWC15[80-229], and C) CDC5L[1-195] alone or as part of the indicated complex. The residue start number (Start), residue end number (End), the charge state (CS), number of potential residues to be deuterated (#D), as well as the retention time (RT) are displayed for every peptide. The time points are labelled for the conditions tested. The relative level of HDX (%HDX, see SI methods) is coloured according to the gradient indicated. The data listed are the average of three independent experiments performed on the same day, with standard deviations (STDEVS) given in the columns on the right.

Figure. S6. Hydrogen deuterium exchanges over time in CTNNBL1 peptides.

Plots presenting the relative deuterium exchange (%HDX, see SI Methods) over time for CTNNBL1[77-563] peptides where the difference was considered significant. The blue curves represent the uncomplexed CTNNBL1, red are CTNNBL1 peptides from the complex of CWC15[80-229] and His-CTNNBL1[77-563], while the green curve is the exchange for the same peptides in the His-CTNNBL1[77-563]/CWC15[80-229]/CDC5L[1-195] complex. The surface model of CTNNBL1 shows the regions corresponding to the peptides. Individual colour represents non-overlapping peptides. Overlapping peptides are grouped and their position linked by the black lines

Figure S7. CTNNBL1 self-associates into a homo-trimer.

SEC-MALS analysis of full length recombinant human His-CTNNBL1[1-563] (red curve), His-CTNNBL[77-563] (green curve), His-CTNNBL[77-563] in 500 mM NaCl (black curve), and the His-CTNNBL[77-563] in complex CWC15[80-229] (blue curve), with excess CWC15 in the second smaller blue peak. Elution volume is depicted on the x-axis, the calculated molar mass in gram/mol on the left y-axis, and the refractive index in arbitrary units (a.u.) on the right y-axis.

Figure S8. CDC5L abundance is reduced but localisation is normal in CTNNBL1 deficient mouse cells.
A) Immunofluorescence for CDC5L and DAPI staining in 4% paraformaldehyde fixed *CD19-Cre Ctnnbl1*$^{+/flx}$ controls (+/-) and *CD19-Cre Ctnnbl1*$^{-/flx}$ (-/-) resting B cells. The signal in the enlarged box showing a single nucleus has been linearly enhanced in the (-/-) pictures to allow visualisation of the characteristic speckled appearance. B) Two independent experiments show reduced levels of CDC5L and PRP19 protein in asynchronous control (+) and Ctnnbl1 deficient (-) ES cell cultures, compared to β-tubulin. Size markers (kDa) are indicated.

Figure S9. Protein disorder predictions for full length human CWC15, CDC5L and CTNNBL1.

For each region of the protein (aa position) disorder disposition is indicated in the y-axis, with values above 0.5 predicting a disordered secondary protein structure and below structured regions as

predicted by three methods, VSL2b (5) (blue curve), VL3 (6)(red curve), VLXT (7) (grey curve) (http://www.disprot.org/).

Fig. S1

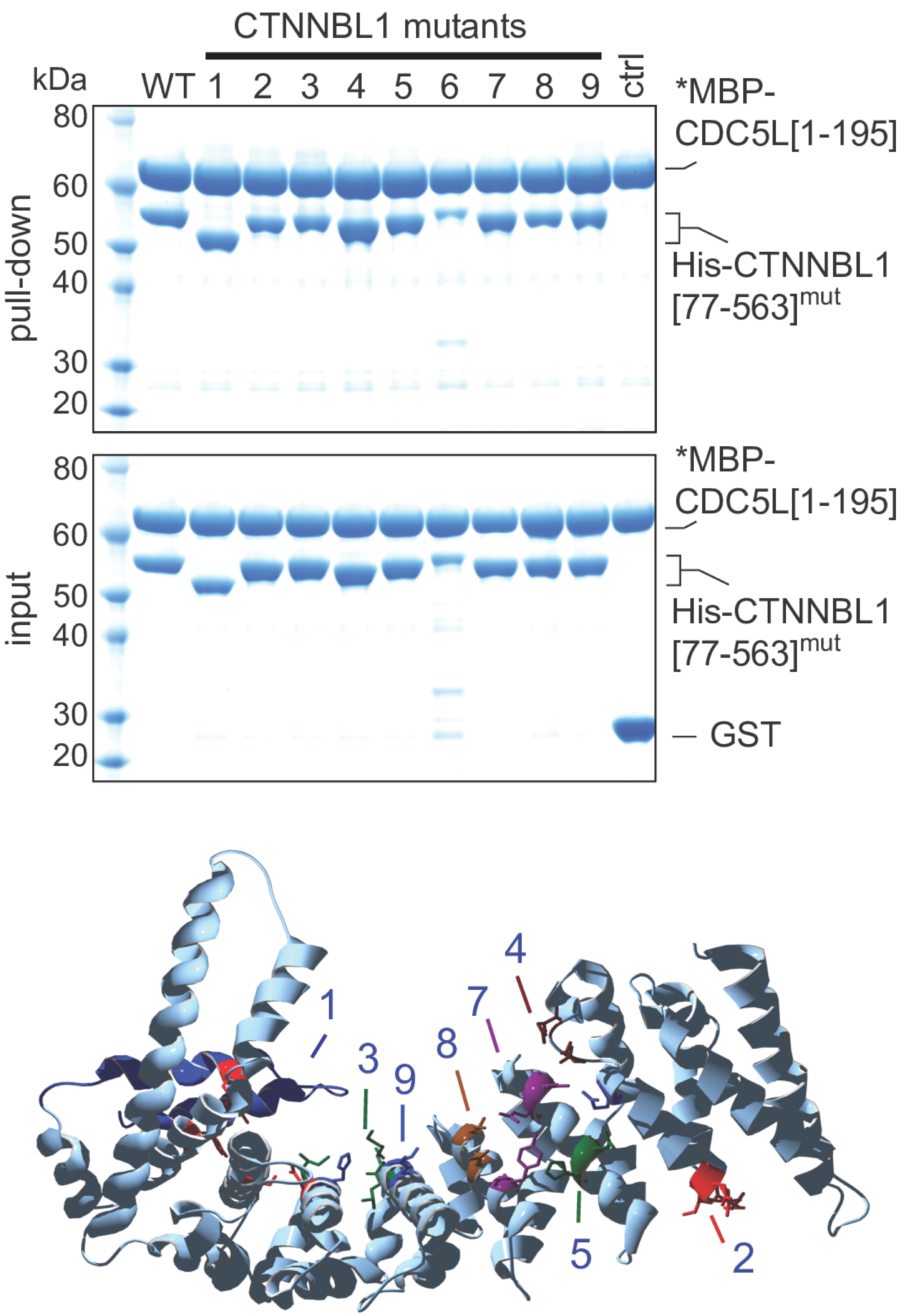
CTNNBL1 mutants
kDa
WT 1 2 3 4 5 6 7 8 9 ctrl
80
60
50
40
30
20
pull-down
input
*MBP-CDC5L[1-195]
His-CTNNBL1 [77-563]mut
GST
1
2
3
4
5
7
8
9

Fig. S2

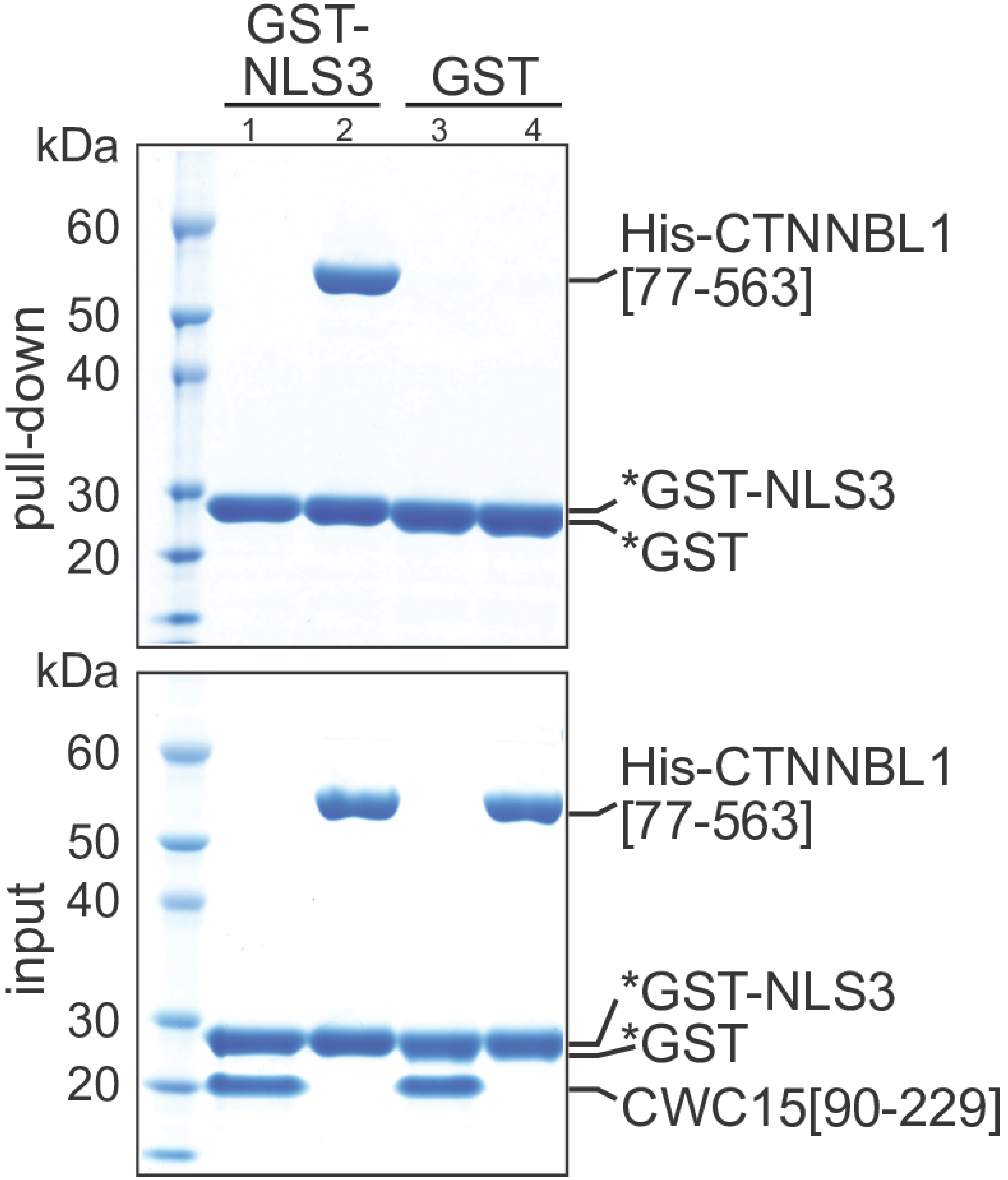

GST-
NLS3
GST
1
2
3
4
kDa
60
50
40
30
20
pull-down
His-CTNNBL1
[77-563]
*GST-NLS3
*GST
kDa
60
50
40
30
20
input
His-CTNNBL1
[77-563]
*GST-NLS3
*GST
CWC15[90-229]

Fig. S3

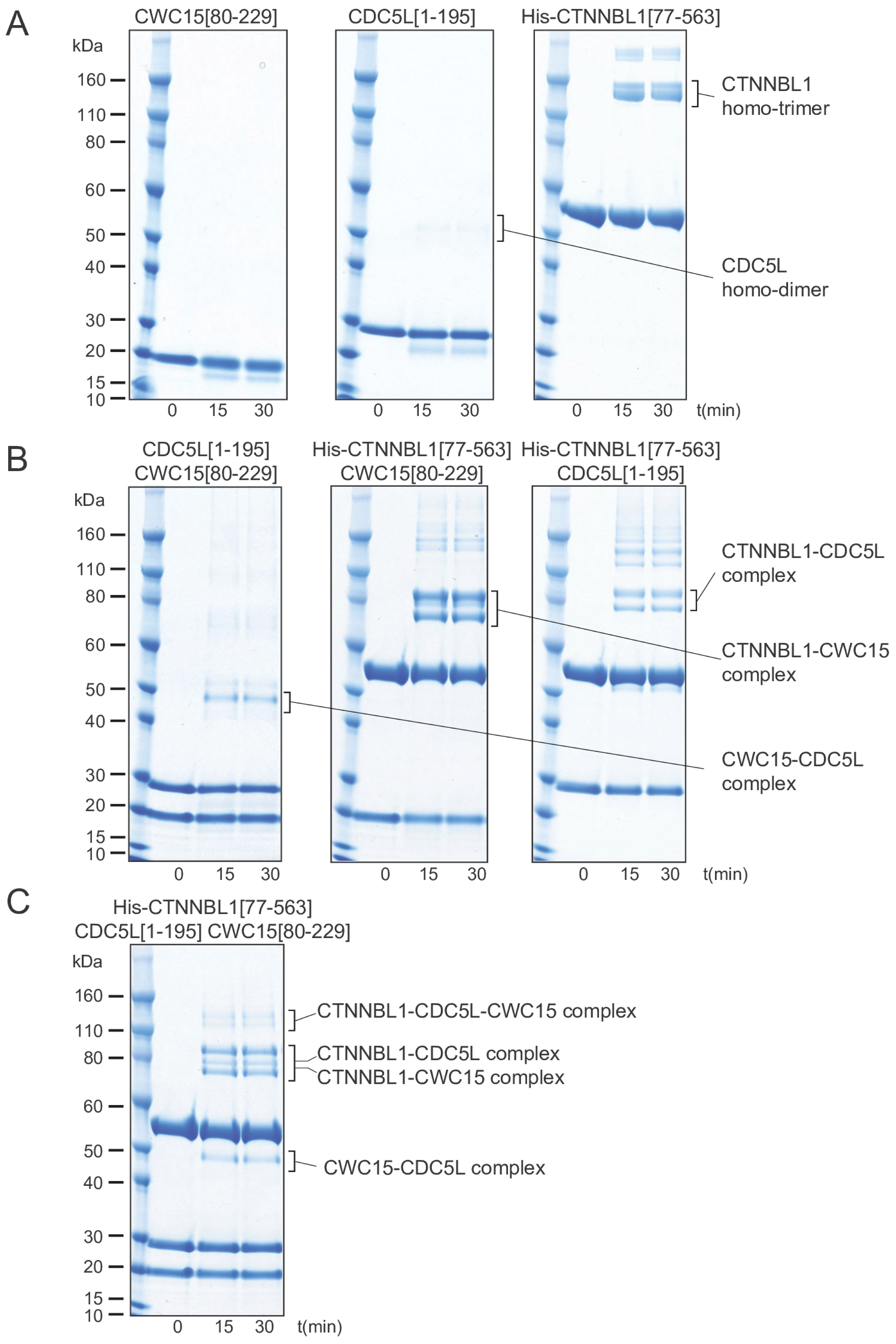
A
CWC15[80-229]
CDC5L[1-195]
His-CTNNBL1[77-563]
kDa
160
110
80
60
50
40
30
20
15
10
0
15
30
t(min)
CTNNBL1
homo-trimer
CDC5L
homo-dimer
B
CDC5L[1-195]
CWC15[80-229]
His-CTNNBL1[77-563]
CWC15[80-229]
His-CTNNBL1[77-563]
CDC5L[1-195]
CTNNBL1-CDC5L
complex
CTNNBL1-CWC15
complex
CWC15-CDC5L
complex
C
His-CTNNBL1[77-563]
CDC5L[1-195] CWC15[80-229]
CTNNBL1-CDC5L-CWC15 complex
CTNNBL1-CDC5L complex
CTNNBL1-CWC15 complex
CWC15-CDC5L complex

Fig. S4

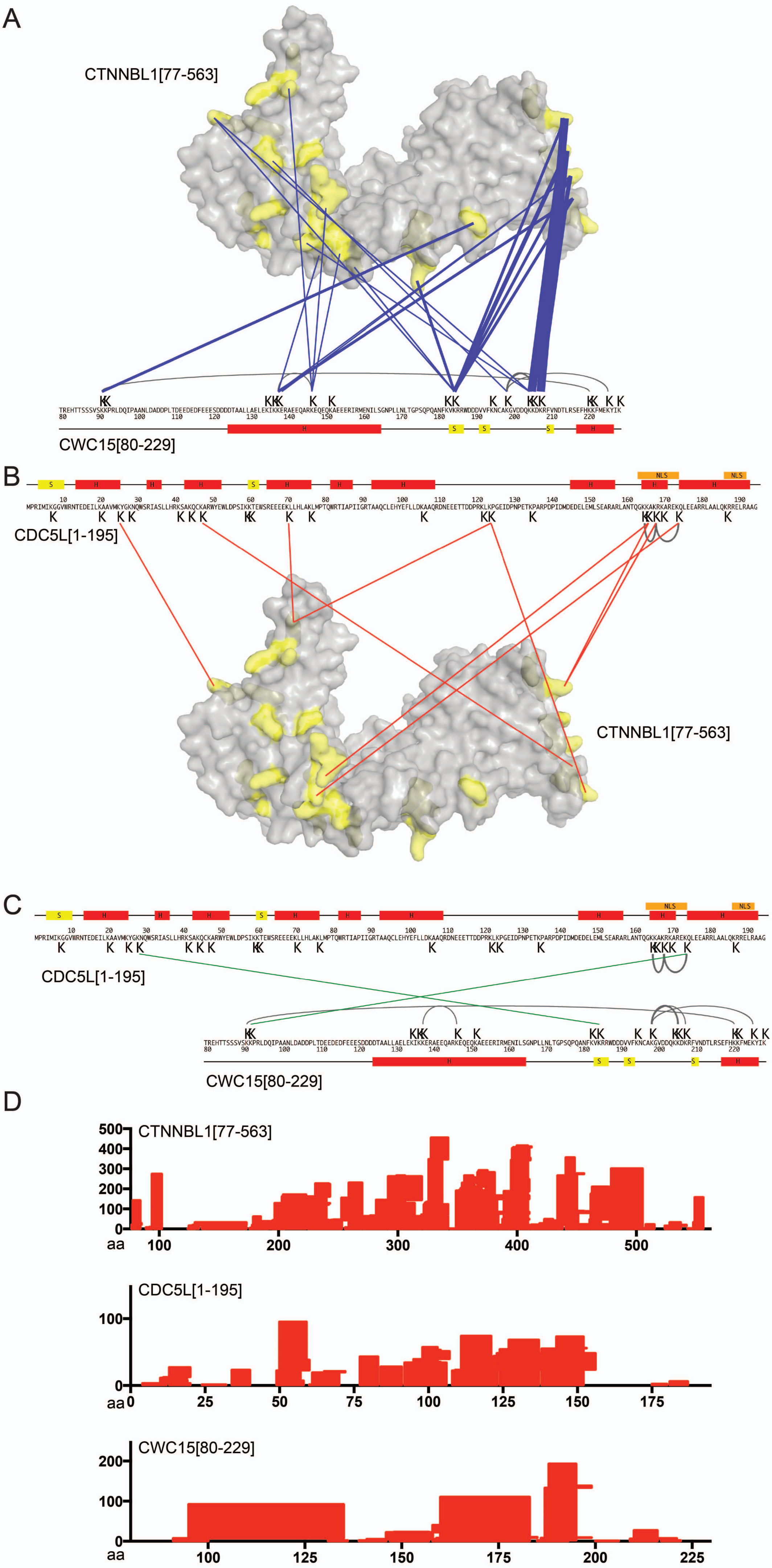
A
CTNNBL1[77-563]
CWC15[80-229]
B
CDC5L[1-195]
CTNNBL1[77-563]
C
CDC5L[1-195]
CWC15[80-229]
D
CTNNBL1[77-563]
CDC5L[1-195]
CWC15[80-229]
aa

Fig. S5-A

					Global HDX levels CTNNBL1					CTNNBL1/CWC15					CTNNBL1/CWC15/CDC5L					STDEVS CTNNBL1					CTNNBL1/CWC15					CTNNBL1/CWC15/CDC5L				
Start	End	CS	#D	RT	3s/4C	3s	30s	300s	3000s	3s/4C	3s	30s	300s	3000s	3s/4C	3s	30s	300s	3000s	3s/4C	3s	30s	300s	3000s	3s/4C	3s	30s	300s	3000s	3s/4C	3s	30s	300s	3000s
69	87	3	17	6.36 - 6.40	6%	9%	15%	24%	30%	6%	9%	16%	25%	28%	7%	11%	18%	26%	29%	1	1	1	1	3	1	1	1	1	0	0	1	1	1	2
69	87	4	17	6.27 - 6.43	7%	9%	15%	24%	31%	6%	9%	14%	24%	26%	6%	10%	17%	24%	28%	1	1	0	1	2	0	0	0	1	1	0	1	1	1	1
69	87	5	17	6.24 - 6.55	6%	10%	16%	24%	30%	6%	9%	15%	24%	28%	6%	10%	17%	25%	28%	2	1	1	1	2	1	0	0	1	0	0	1	0	0	2
69	88	3	18	6.04 - 6.28	7%	9%	15%	25%	33%	6%	9%	14%	24%	30%	6%	10%	17%	26%	32%	1	1	1	0	1	1	0	0	1	1	0	1	0	0	2
69	88	4	18	6.08 - 6.27	7%	10%	16%	26%	34%	6%	9%	15%	25%	31%	6%	10%	17%	26%	32%	2	1	0	0	1	1	0	0	1	0	0	1	0	0	2
69	88	5	18	6.11 - 6.28	6%	9%	15%	25%	34%	6%	9%	15%	25%	31%	6%	11%	18%	26%	33%	1	1	1	1	1	0	0	0	1	0	0	1	0	0	2
88	98	2	9	3.76 - 3.96	1%	2%	10%	32%	47%	2%	2%	3%	6%	22%	5%	2%	3%	5%	19%	0	0	1	1	3	1	0	0	0	1	1	0	1	0	2
88	98	3	9	3.76 - 3.96	1%	2%	10%	32%	47%	1%	1%	2%	5%	23%	2%	2%	3%	5%	20%	0	0	1	1	3	1	0	0	0	1	0	0	1	1	2
89	99	2	9	4.75 - 4.93	1%	2%	10%	33%	48%	1%	2%	2%	5%	18%	2%	2%	2%	4%	15%	0	0	1	1	4	0	0	0	0	0	0	0	1	0	n/a
99	110	3	8	8.35 - 8.55	9%	15%	25%	50%	56%	3%	8%	15%	17%	25%	3%	8%	15%	16%	21%	3	1	1	1	0	0	0	0	1	1	0	1	1	0	2
99	114	3	12	10.00 - 10.09	20%	26%	39%	62%	68%	14%	19%	26%	32%	39%	14%	18%	26%	31%	36%	3	1	1	1	2	1	0	0	0	0	0	1	1	0	2
100	110	2	7	7.59 - 7.79	10%	18%	30%	57%	62%	3%	10%	18%	22%	31%	3%	10%	19%	20%	27%	3	1	1	1	2	0	0	0	1	1	0	0	1	0	2
100	110	3	7	7.61 - 7.74	11%	19%	31%	58%	63%	4%	10%	19%	23%	31%	3%	10%	19%	21%	28%	4	1	1	1	3	0	0	0	1	1	0	0	1	0	2
100	115	2	12	9.84 - 10.07	20%	26%	39%	62%	69%	14%	19%	26%	32%	39%	14%	18%	26%	31%	36%	3	1	1	1	2	1	0	0	0	0	0	1	0	0	1
100	115	3	12	10.02 - 10.10	20%	26%	39%	61%	68%	14%	19%	26%	32%	38%	14%	18%	26%	30%	36%	3	1	1	1	2	1	0	0	0	0	0	1	1	0	2
118	124	1	5	9.37 - 9.59	1%	2%	2%	2%	15%	1%	2%	3%	2%	2%	0%	3%	2%	2%	3%	1	1	0	0	2	0	1	1	0	0	0	1	1	1	1
118	133	3	13	11.09 - 11.38	2%	3%	10%	13%	31%	2%	3%	10%	16%	29%	2%	6%	15%	21%	31%	2	0	1	0	1	0	0	0	0	1	0	0	0	1	1
120	124	1	3	7.18 - 7.44	0%	1%	1%	2%	13%	0%	1%	3%	3%	3%	0%	1%	2%	2%	5%	1	1	0	2	1	1	0	1	1	2	1	0	0	1	1
120	133	2	11	9.91 - 10.09	2%	3%	10%	14%	31%	2%	2%	10%	18%	34%	3%	6%	17%	22%	37%	2	0	0	0	1	1	0	0	0	1	1	1	1	0	1
125	133	2	6	8.49 - 8.78	2%	3%	5%	8%	26%	2%	2%	5%	14%	29%	2%	4%	12%	18%	36%	1	0	0	1	2	0	0	0	0	1	0	0	0	0	1
125	140	3	13	11.63 - 11.89	3%	3%	5%	13%	34%	3%	3%	5%	15%	36%	3%	3%	8%	20%	40%	1	1	0	0	1	1	0	0	1	1	0	0	1	2	2
131	140	2	8	11.79 - 11.96	2%	3%	4%	16%	36%	4%	5%	6%	19%	36%	5%	5%	9%	21%	40%	0	0	1	1	0	1	1	1	1	1	0	1	2	0	n/a
141	160	2	18	11.50 - 11.66	4%	8%	18%	24%	29%	2%	9%	19%	23%	28%	2%	9%	20%	23%	30%	4	0	1	1	1	0	0	0	1	0	0	0	0	0	1
147	158	2	10	7.88 - 8.00	2%	3%	8%	10%	14%	2%	4%	8%	9%	12%	1%	3%	8%	8%	11%	2	0	1	1	2	0	0	0	0	0	0	0	1	0	1
147	160	2	12	8.95 - 9.11	3%	4%	11%	15%	19%	2%	6%	12%	13%	17%	1%	5%	11%	12%	16%	3	0	0	0	1	0	0	0	0	0	0	0	0	0	1
147	161	2	13	9.53 - 9.71	3%	4%	11%	15%	19%	2%	5%	11%	12%	15%	2%	6%	11%	11%	15%	3	0	0	0	1	1	0	0	0	0	0	2	0	0	1
148	159	2	10	7.89 - 8.00	2%	3%	8%	10%	14%	2%	4%	8%	9%	12%	1%	3%	8%	8%	11%	2	0	1	1	2	0	0	0	0	0	0	0	1	0	1
160	164	1	3	8.38 - 8.63	2%	3%	4%	10%	15%	4%	6%	6%	8%	15%	6%	6%	6%	6%	12%	1	1	1	1	2	1	0	1	0	0	1	1	3	2	0
161	176	2	14	11.38 - 11.51	2%	10%	23%	30%	37%	2%	10%	24%	30%	35%	2%	11%	24%	29%	37%	0	0	1	0	1	0	0	0	0	1	0	1	0	0	2
165	170	1	4	6.27 - 6.55	0%	1%	1%	0%	0%	0%	0%	1%	1%	1%	1%	0%	1%	0%	1%	0	0	0	0	1	0	0	0	2	1	1	0	0	0	1
165	173	1	7	8.18 - 8.45	3%	2%	2%	8%	24%	3%	3%	13%	18%	27%	2%	8%	17%	19%	32%	4	1	1	1	1	0	0	3	0	4	1	1	3	2	1
165	181	2	15	8.52 - 8.71	2%	6%	9%	14%	26%	3%	7%	15%	23%	25%	4%	11%	21%	21%	27%	0	1	1	1	3	0	0	1	1	1	0	1	1	1	n/a
171	184	2	12	8.30 - 8.45	3%	9%	15%	24%	40%	5%	13%	24%	36%	42%	9%	22%	36%	43%	50%	0	1	1	1	2	0	2	2	2	3	0	1	0	0	4
183	196	2	12	11.28 - 11.44	1%	1%	2%	6%	19%	1%	1%	2%	7%	22%	0%	1%	5%	11%	28%	0	0	0	0	0	0	0	0	0	0	0	1	1	0	2
185	196	2	10	9.96 - 10.20	1%	1%	1%	5%	22%	1%	1%	2%	8%	28%	1%	1%	3%	12%	35%	0	0	0	0	0	0	0	0	0	0	0	0	0	0	1
185	197	2	11	11.23 - 11.38	0%	0%	1%	4%	21%	0%	1%	1%	6%	22%	-1%	1%	2%	8%	27%	0	0	0	0	0	0	0	0	0	0	0	0	0	0	n/a
197	206	2	8	7.64 - 7.81	1%	1%	4%	10%	26%	2%	2%	4%	11%	27%	2%	2%	4%	8%	26%	1	0	0	1	1	0	1	1	0	1	0	1	1	1	3
197	220	3	22	9.16 - 9.30	7%	9%	18%	34%	48%	6%	9%	16%	28%	44%	6%	8%	15%	23%	42%	2	0	1	0	1	0	0	0	1	1	0	0	0	0	0
202	206	1	3	4.19 - 4.45	2%	3%	6%	12%	19%	4%	6%	7%	11%	18%	7%	7%	9%	6%	12%	1	1	0	1	3	1	2	0	0	1	1	1	4	0	2
202	219	3	16	7.47 - 7.64	9%	12%	22%	39%	48%	7%	11%	19%	31%	43%	7%	10%	18%	26%	40%	3	0	1	0	1	0	0	0	0	0	0	1	0	0	2
202	220	3	17	7.42 - 7.61	9%	11%	21%	38%	48%	7%	10%	18%	30%	42%	7%	10%	17%	24%	39%	2	0	0	1	1	0	0	0	1	1	0	1	1	0	2
207	219	2	11	5.36 - 5.89	10%	13%	20%	34%	46%	9%	12%	17%	25%	38%	8%	11%	17%	20%	34%	2	0	1	0	2	0	0	0	1	0	0	0	1	0	2
207	219	3	11	5.37 - 5.92	10%	13%	20%	34%	46%	9%	12%	17%	26%	38%	9%	12%	18%	21%	35%	2	0	1	1	2	1	0	1	1	0	0	0	1	1	2
221	225	1	3	6.04 - 6.38	1%	1%	2%	0%	-1%	2%	3%	2%	2%	2%	2%	1%	2%	1%	0%	2	1	1	1	1	2	2	0	1	2	2	0	1	1	1
221	227	1	5	5.70 - 6.06	1%	2%	2%	2%	3%	2%	2%	2%	4%	7%	1%	2%	2%	2%	5%	0	0	0	0	1	1	0	0	1	1	0	0	1	1	0
226	232	1	4	7.69 - 7.82	12%	20%	25%	36%	44%	8%	19%	26%	43%	48%	8%	20%	28%	38%	49%	7	1	1	1	2	1	1	1	4	1	0	1	2	1	7
226	232	2	4	7.68 - 7.82	14%	20%	25%	38%	45%	10%	21%	27%	41%	47%	10%	21%	29%	40%	49%	7	1	1	1	2	0	0	1	1	1	1	1	2	0	5
227	232	1	3	7.65 - 7.88	19%	27%	35%	44%	52%	12%	24%	34%	47%	54%	14%	25%	34%	48%	57%	10	3	1	5	3	2	1	4	1	1	1	1	1	2	4
228	232	1	2	7.67 - 7.84	11%	28%	34%	49%	60%	17%	28%	35%	54%	61%	10%	27%	34%	53%	57%	1	2	1	2	2	1	1	2	3	2	2	1	1	2	1
233	260	3	25	10.87 - 11.03	11%	15%	24%	33%	41%	10%	14%	25%	35%	39%	9%	14%	25%	32%	40%	3	1	2	1	3	1	1	0	2	1	1	1	1	0	2
233	260	4	25	10.86 - 11.12	11%	15%	24%	33%	41%	9%	14%	23%	34%	38%	9%	14%	22%	29%	38%	3	1	2	1	3	0	0	1	1	1	0	0	1	0	2
246	260	4	12	6.64 - 6.81	18%	24%	30%	37%	42%	18%	23%	29%	37%	40%	16%	21%	27%	29%	40%	2	2	2	1	2	1	1	1	1	1	0	1	1	1	3
261	266	1	4	8.55 - 8.80	3%	2%	3%	3%	6%	5%	3%	4%	6%	7%	5%	4%	5%	2%	8%	2	1	0	0	1	0	1	2	4	2	n/a	0	2	2	n/a
268	280	2	11	9.27 - 9.46	2%	3%	3%	6%	12%	2%	4%	6%	10%	14%	2%	3%	4%	7%	11%	0	0	0	1	1	1	1	0	1	1	0	1	1	2	1
268	283	2	14	10.30 - 10.50	1%	2%	3%	6%	13%	1%	1%	3%	7%	14%	1%	1%	3%	5%	11%	0	0	0	0	0	0	0	0	0	1	0	0	0	0	2
270	275	1	4	3.92 - 4.15	30%	41%	53%	54%	58%	24%	39%	49%	56%	54%	23%	36%	50%	53%	56%	9	3	3	1	7	2	2	2	1	1	2	2	2	2	6
270	280	2	9	7.97 - 8.13	2%	3%	4%	7%	9%	2%	3%	4%	8%	9%	1%	2%	4%	7%	9%	1	1	1	1	1	1	1	0	1	1	0	0	1	0	1
270	283	2	12	9.61 - 9.84	2%	1%	3%	5%	10%	2%	1%	2%	6%	12%	2%	2%	2%	5%	10%	0	0	1	0	1	1	0	0	0	0	1	1	0	0	1
270	287	2	16	9.79 - 10.27	1%	1%	4%	12%	19%	1%	1%	4%	13%	19%	1%	1%	4%	12%	18%	0	0	0	1	2	0	0	0	0	0	0	0	0	0	2
270	289	2	18	11.14 - 11.33	1%	1%	2%	8%	16%	1%	1%	3%	10%	16%	0%	1%	3%	9%	16%	0	0	0	0	2	1	0	0	0	1	0	0	0	0	2
276	283	2	6	9.34 - 9.49	2%	2%	2%	2%	9%	2%	2%	2%	3%	12%	1%	1%	2%	2%	11%	0	1	0	0	1	2	1	1	0	1	0	0	1	0	1
284	289	1	4	9.63 - 9.80	1%	1%	5%	18%	30%	1%	2%	5%	20%	28%	2%	3%	7%	19%	30%	0	1	1	2	2	1	0	0	0	1	1	1	2	1	4
288	295	1	6	9.82 - 10.05	1%	2%	11%	17%	25%	1%	2%	13%	19%	26%	0%	4%	15%	17%	27%	1	0	0	0	1	0	0	0	1	1	0	1	0	0	1
288	313	4	23	9.84 - 10.12	5%	7%	11%	15%	23%	5%	8%	14%	18%	26%	3%	6%	13%	15%	19%	1	1	1	1	2	1	1	1	0	1	0	0	1	1	1
290	309	3	17	7.21 - 7.54	8%	12%	20%	25%	30%	7%	13%	22%	26%	32%	6%	9%	21%	23%	26%	2	1	1	1	2	1	1	2	1	0	1	1	1	1	2
290	313	4	21	9.06 - 9.29	5%	7%	11%	14%	21%	8%	10%	15%	18%	27%	6%	8%	13%	15%	20%	1	1	1	1	2	2	0	1	1	1	0	1	1	1	n/a
296	306	2	8	3.39 - 3.70	22%	27%	31%	33%	38%	21%	26%	32%	34%	38%	19%	25%	32%	31%	34%	3	0	1	1	2	1	1	1	1	0	1	1	1	0	3
321	338	3	16	8.95 - 9.06	6%	8%	14%	24%	32%	6%	8%	16%	27%	34%	5%	8%	16%	24%	33%	1	0	1	1	3	0	0	1	1	2	0	0	1	1	3
323	338	3	14	8.11 - 8.39	6%	8%	14%	25%	34%	6%	9%	17%	29%	37%	5%	8%	18%	26%	36%	1	0	1	1	3	0	0	0	1	1	0	0	1	1	3
324	338	3	13	8.06 - 8.38	7%	10%	17%	31%	42%	7%	10%	22%	35%	43%	7%	10%	21%	31%	45%	1	1	1	2	2	0	0	1	1	1	0	1	0	1	n/a
342	353	3	10	3.56 - 3.75	19%	25%	30%	36%	40%	16%	24%	29%	38%	37%	8%	21%	27%	32%	39%	4	1	2	1	3	1	1	1	2	2	1	2	1	1	4
342	354	2	11	4.48 - 4.63	11%	15%	19%	24%	32%	10%	14%	18%	26%	30%	5%	12%	16%	19%	26%	2	1	1	1	3	1	1	1	1	1	0	1	1	1	3
342	354	3	11	4.49 - 4.62	11%	15%	19%	24%	32%	10%	14%	18%	26%	30%	5%	13%	17%	20%	27%	2	1	2	1	3	1	1	1	2	1	0	1	1	1	4
342	378	5	34	8.76 - 8.92	3%	5%	8%	10%	16%	4%	5%	7%	10%	15%	2%	5%	7%	8%	13%	1	1	1	0	2	0	0	0	0	1	0	0	1	0	1
342	379	5	35	9.47 - 9.74	4%	7%	10%	12%	18%	3%	5%	9%	11%	16%	2%	4%	7%	9%	14%	1	1	1	0	2	0	0	0	0	1	0	0	1	0	1
342	379	6	35	9.55 - 9.76	4%	6%	9%	12%	17%	3%	5%	8%	11%	16%	2%	4%	7%	9%	14%	1	0	1	1	2	0	0	0	0	1	0	0	1	0	1
343	353	3	9	3.63 - 3.81	17%	24%	27%	31%	37%	15%	22%	25%	32%	37%	7%	18%	22%	25%	33%	2	2	2	2	2	2	1	1	1	1	0	1	1	2	5
343	354	3	10	3.92 - 4.50	13%	19%	22%	26%	33%	12%	15%	21%	27%	31%	6%	15%	17%	20%	28%	3	2	2	1	2	1	1	1	1	1	0	2	1	1	2
343	379	6	34	9.54 - 9.78	4%	6%	9%	12%	18%	4%	5%	8%	11%	16%	2%	4%	7%	9%	14%	1	0	1	1	2	0	0	0	0	0	0	0	1	0	1
344	354	2	9	3.65 - 3.82	17%	23%	26%	30%	37%	15%	21%	23%	32%	36%	7%	17%	21%	24%	29%	2	2	2	1	2	2	1	1	1	1	0	2	2	3	3
344	354	3	9	3.63 - 3.82	17%	24%	27%	31%	38%	15%	22%	25%	33%	36%	7%	18%	22%	25%	33%	2	2	2	2	2	2	1	1	1	1	0	1	1	2	5
354	379	3	23	10.33 - 10.62	3%	5%	8%	10%	14%	2%	3%	7%	9%	13%	1%	3%	6%	8%	13%	1	0	1	0	1	0	0	0	0	1	0	0	1	0	1
354	379	4	23	10.35 - 10.59	3%	5%	8%	11%	15%	2%	4%	7%	10%	14%	1%	3%	6%	8%	13%	1	0	1	0	1	0	0	0	0	0	0	0	1	0	1
355	373	3	16	7.41 - 7.61	9%	12%	22%	40%	51%	8%	11%	19%	32%	45%	7%	10%	18%	26%	42%	2	0	0	1	2	0	0	0	0	0	0	1	1	0	2
355	377	4	20	9.11 - 9.24	2%	4%	7%	8%	13%	3%	4%	6%	8%	13%	2%	4%	6%	7%	13%	0	0	1	1	2	0	0	0	0	1	0	0	1	0	3
355	378	4	21	8.95 - 9.07	3%	4%	7%	10%	21%	2%	3%	7%	11%	13%	1%	3%	7%	10%	13%	1	1	1	0	5	1	0	0	5	0	0	1	1	3	1
355	379	2	22	10.16 - 10.61	3%	6%	10%	12%	17%	2%	4%	8%	11%	16%	2%	3%	7%	10%	15%	1	0	1	0	1	0	0	0	0	0	0	0	1	0	1
355	379	3	22	10.18 - 10.42	3%	6%	9%	12%	16%	2%	4%	8%	10%	15%	2%	3%	7%	9%	15%	1	0	1	0	1	0	0	0	0	1	0	0	1	0	1
355	379	4	22	10.16 - 10.41	3%	6%	10%	12%	17%	3%	4%	8%	11%	16%	2%	3%	7%	10%	15%	1	0	1	0	1	0	0	0	0	0	0	0	1	0	1
371	379	2	7	10.30 - 10.56	4%	8%	13%	19%	27%	2%	5%	12%	16%	26%	2%	3%	6%	13%	22%	2	0	0	1	1	1	0	1	0	1	0	0	1	0	1
378	386	2	6	11.77 - 11.97	2%	3%	7%	11%	30%	1%	2%	5%	10%	19%	1%	1%	3%	6%	13%	1	0	1	0	2	0	0	0	1	1	0	0	1	0	1
379	386	2	5	11.69 - 11.91	1%	1%	2%	5%	22%	1%	1%	1%	3%	9%	1%	1%	1%	1%	4%	0	0	0	0	1	0	0	0	0	1	0	0	0	0	0
380	386	1	4	11.37 - 11.58	-1%	1%	1%	4%	22%	-1%	0%	1%	0%	5%	-1%	0%	1%	0%	1%	0	0	0	0	1	0	0	0	0	0	0	0	1	0	1
380	386	2	4	11.41 - 11.62	1%	1%	1%	5%	23%	1%	1%	1%	2%	7%	1%	1%	1%	1%	2%	0	0	0	0	1	0	0	0	0	0	0	0	1	0	0
380	411	6	28	7.76 - 8.17	12%	14%	15%	15%	22%	12%	13%	14%	15%	17%	9%	12%	14%	14%	15%	1	1	1	1	3	1	1	0	1	0	0	1	1	1	2
387	411	3	22	4.92 - 5.23	14%	16%	17%	18%	22%	14%	15%	17%	19%	20%	10%	14%	16%	16%	17%	1	1	2	0	1	1	0	1	1	1	1	1	1	1	1
387	411	4	22	4.92 - 5.47	13%	17%	18%	17%	22%	14%	16%	17%	19%	21%	10%	14%	17%	17%	18%	1	2	1	1	3	1	1	1	1	1	0	1	1	0	2
387	411	5	22	4.92 - 5.37	13%	16%	16%	17%	22%	14%	15%	17%	18%	20%	10%	13%	16%	16%	17%	1	2	0	0	2	1	0	1	1	0	1	1	1	0	1
415	426	2	10	4.21 - 4.38	12%	17%	27%	31%	34%	11%	16%	24%	30%	33%	11%	17%	25%	28%	35%	2	1	1	1	0	0	0	1	1	1	1	1	0	1	3
415	441	4	25	6.39 - 6.69	2%	4%	9%	15%	20%	2%	3%	7%	14%	18%	2%	3%	8%	13%	19%	1	0	1	1	2	1	0	0	1	0	0	0	0	1	1
415	441	5	25	6.47 - 6.57	2%	3%	9%	15%	20%	2%	3%	7%	15%	17%	2%	3%	8%	13%	18%	1	0	1	1	3	0	0	0	1	1	0	0	1	0	1
415	441	6	25	6.49 - 6.71	2%	3%	9%	15%	19%	2%	3%	7%	14%	17%	2%	3%	8%	14%	18%	1	0	1	1	2	0	0	0	1	1	0	0	1	0	1
427	436	2	8	4.95 - 5.47	11%	11%	23%	37%	43%	9%	9%	18%	35%	37%	8%	10%	19%	35%	40%	2	1	3	1	5	1	0	2	1	1	0	1	1	1	5
427	441	2	13	6.44 - 6.78	2%	3%	9%	20%	23%	4%	5%	9%	22%	27%	3%	4%	10%	19%	25%	1	0	0	0	1	1	0	0	1	0	0	0	1	0	1
427	441	3	13	6.44 - 6.83	2%	3%	9%	20%	23%	4%	4%	9%	22%	27%	3%	4%	9%	19%	25%	1	0	0	0	1	1	0	0	1	1	0	0	1	0	1
427	441	4	13	6.47 - 6.77	2%	3%	9%	20%	24%	4%	5%	9%	22%	27%	3%	4%	10%	20%	25%	1	0	0	1	1	1	0	0	1	0	0	0	0	0	1
428	441	3	12	6.44 - 6.59	2%	3%	10%	21%	24%	5%	5%	9%	23%	28%	4%	4%	10%	21%	28%	1	0	0	1	1	1	0	0	1	1	0	0	0	0	2
431	441	3	9	5.29 - 5.74	2%	3%	10%	21%	25%	3%	4%	7%	17%	23%	3%	3%	7%	14%	22%	1	1	0	0	1	1	0	0	1	1	1	1	0	0	1
442	451	2	8	9.13 - 9.29	2%	4%	13%	16%	16%	2%	4%	14%	16%	16%	n/a	4%	14%	17%	19%	0	0	0	0	1	0	0	0	1	0	n/a	0	0	1	1
442	481	5	38	8.93 - 9.07	9%	36%	56%	55%	58%	6%	35%	55%	56%	56%	6%	22%	50%	54%	57%	4	0	1	1	2	1	0	1	1	1	0	1	1	0	3
445	453	2	7	7.55 - 7.78	4%	8%	32%	40%	42%	3%	8%	32%	38%	37%	6%	9%	15%	17%	18%	0	0	3	1	3	1	0	0	1	1	1	1	0	1	2
452	480	3	27	5.87 - 6.12	8%	39%	55%	54%	55%	7%	38%	53%	55%	54%	8%	23%	51%	54%	56%	0	1	2	0	3	1	0	1	1	0	0	1	1	1	4
452	481	3	28	7.18 - 7.34	8%	41%	58%	57%	58%	8%	39%	57%	58%	57%	8%	25%	53%	57%	59%	0	2	1	1	3	1	0	0	1	1	0	1	2	1	2
453	481	3	27	7.03 - 7.26	8%	42%	60%	59%	59%	8%	40%	58%	59%	58%	8%	25%	53%	58%	60%	0	1	2	1	2	1	1	0	0	0	0	1	1	0	4
481	490	2	8	9.02 - 9.32	2%	14%	42%	49%	52%	1%	13%	42%	50%	49%	4%	6%	17%	34%	54%	0	1	3	1	4	0	0	1	2	1	1	2	1	1	n/a
481	490	3	8	9.17 - 9.20	1%	15%	45%	52%	54%	1%	14%	44%	53%	51%	1%	3%	16%	33%	52%	1	1	3	2	5	0	0	2	3	2	0	0	0	1	5
482	490	2	7	7.54 - 7.70	1%	17%	46%	52%	54%	1%	16%	44%	52%	49%	1%	4%	19%	34%	49%	1	1	2	2	4	0	0	1	2	2	0	0	1	1	5
483	490	2	6	9.12 - 9.34	1%	22%	54%	60%	63%	2%	19%	52%	61%	60%	2%	4%	23%	43%	63%	1	2	3	2	5	1	1	2	2	2	0	0	0	2	3
491	500	2	8	11.25 - 11.45	0%	0%	0%	0%	2%	0%	0%	0%	0%	2%	0%	0%	0%	0%	0%	0	0	0	0	0	0	0	0	0	0	0	0	0	0	0
493	498	1	4	9.66 - 9.95	0%	-1%	0%	-1%	1%	0%	-1%	0%	0%	1%	1%	0%	0%	-1%	-1%	1	0	0	0	1	1	1	0	1	1	1	0	0	1	1
499	504	1	4	7.67 - 7.84	8%	14%	18%	25%	30%	10%	14%	19%	27%	33%	6%	13%	19%	26%	29%	4	2	0	1	1	2	0	1	1	2	3	1	1	0	1
499	521	3	20	10.23 - 10.41	10%	15%	25%	36%	48%	9%	15%	26%	37%	47%	9%	15%	23%	32%	45%	2	1	1	0	2	1	0	0	0	1	0	1	1	1	3
503	519	3	14	7.81 - 8.24	9%	13%	19%	29%	41%	10%	13%	19%	32%	40%	11%	13%	19%	27%	38%	1	1	1	2	2	0	1	1	1	2	0	1	0	1	3
503	521	3	16	9.40 - 9.50	9%	13%	25%	37%	50%	8%	13%	25%	38%	49%	7%	13%	24%	35%	48%	2	1	1	1	4	1	0	1	1	1	0	1	0	1	2
505	521	2	14	8.98 - 9.10	7%	11%	24%	36%	52%	6%	12%	25%	38%	52%	5%	11%	24%	36%	51%	2	1	1	2	4	1	0	0	2	1	0	1	1	1	3
522	535	4	12	4.75 - 5.02	16%	20%	27%	53%	63%	15%	20%	30%	57%	60%	14%	18%	22%	36%	60%	1	0	1	1	3	1	0	0	1	1	1	1	0	1	4
522	535	5	12	4.78 - 4.97	16%	20%	28%	52%	62%	15%	20%	30%	56%	59%	15%	18%	22%	35%	60%	2	0	1	0	2	1	1	1	2	1	1	1	0	1	4
522	539	3	16	7.11 - 7.30	12%	17%	30%	61%	71%	12%	17%	34%	66%	68%	11%	15%	21%	38%	65%	2	1	1	0	4	1	0	1	1	1	0	1	1	0	3
522	563	6	39	10.09 - 10.37	13%	20%	38%	58%	64%	12%	19%	40%	59%	60%	11%	16%	29%	47%	63%	2	1	2	0	3	1	1	1	1	1	1	1	1	0	3
526	535	2	8	4.59 - 4.75	2%	4%	10%	30%	41%	2%	3%	12%	35%	39%	2%	3%	5%	17%	40%	0	0	1	1	3	1	0	0	2	1	0	0	1	1	5
526	535	3	8	4.64 - 4.65	2%	4%	10%	30%	41%	2%	3%	12%	35%	39%	1%	2%	4%	16%	38%	0	0	1	1	3	0	0	0	1	1	0	0	0	1	3
526	548	3	20	8.32 - 8.55	17%	22%	36%	57%	64%	15%	20%	36%	59%	61%	12%	15%	24%	41%	60%	2	1	1	1	3	1	1	1	0	1	0	0	1	1	3
536	548	2	10	8.12 - 8.35	27%	33%	41%	41%	42%	26%	31%	39%	42%	40%	22%	25%	33%	38%	42%	3	2	2	1	3	2	1	2	2	1	2	2	1	1	4
536	559	3	21	8.04 - 8.34	11%	15%	31%	43%	46%	10%	14%	32%	45%	44%	8%	11%	22%	38%	47%	2	1	2	1	3	1	1	1	1	1	0	1	1	1	1
540	548	2	6	7.47 - 7.64	34%	42%	44%	42%	43%	32%	38%	40%	41%	40%	32%	39%	41%	40%	42%	2	2	2	1	2	2	1	1	1	1	1	2	1	1	3
540	559	4	17	7.81 - 8.15	7%	11%	26%	42%	44%	6%	10%	27%	44%	43%	7%	12%	21%	36%	38%	1	1	2	1	3	0	0	0	1	1	1	1	3	1	3
549	559	2	9	7.18 - 7.61	1%	5%	30%	60%	63%	1%	5%	34%	63%	63%	1%	3%	19%	53%	65%	1	1	1	1	2	0	0	3	1	0	0	0	1	1	3
549	559	3	9	7.25 - 7.43	2%	5%	31%	60%	64%	2%	5%	33%	60%	61%	2%	3%	19%	51%	65%	1	0	1	2	4	0	0	1	1	2	0	0	0	2	4
549	560	3	10	8.84 - 9.12	3%	6%	35%	65%	68%	2%	6%	40%	67%	67%	1%	4%	25%	58%	71%	1	1	2	0	3	1	1	3	1	0	0	1	1	1	4
549	563	2	13	10.45 - 10.69	11%	18%	48%	72%	75%	9%	18%	51%	73%	74%	8%	16%	38%	66%	76%	4	1	1	0	2	1	0	0	0	0	0	1	3	1	3
549	563	3	13	10.45 - 10.70	11%	18%	48%	72%	75%	8%	18%	52%	74%	74%	9%	16%	42%	68%	77%	3	0	2	1	1	1	0	1	0	0	0	1	1	1	5

Relative deuteration % 10% 20% 30% 40% 50% 60% 70% 80%

Fig. S5-B

Global HDX levels

Start	End	CS	#D	RT	CWC15 3s/4C	CWC15 3s	CWC15 30s	CWC15 300s	CWC15 3000s	CWC15/CTNNBL1 3s/4C	CWC15/CTNNBL1 3s	CWC15/CTNNBL1 30s	CWC15/CTNNBL1 300s	CWC15/CTNNBL1 3000s	CWC15/CDC5L 3s/4C	CWC15/CDC5L 3s	CWC15/CDC5L 30s	CWC15/CDC5L 300s	CWC15/CDC5L 3000s	CWC15/CDC5L/CTNNBL1 3s/4C	CWC15/CDC5L/CTNNBL1 3s	CWC15/CDC5L/CTNNBL1 30s	CWC15/CDC5L/CTNNBL1 300s	CWC15/CDC5L/CTNNBL1 3000s
75	95	3	18	4.36 - 4.60	30%	29%	30%	28%	29%	30%	27%	27%	28%	26%	27%	28%	27%	27%	28%	27%	26%	26%	26%	27%
75	95	4	18	4.33 - 4.64	30%	29%	30%	28%	29%	30%	27%	27%	28%	26%	27%	28%	28%	28%	28%	27%	27%	27%	26%	27%
75	95	5	18	4.32 - 4.65	30%	29%	30%	28%	29%	30%	28%	27%	29%	27%	28%	28%	28%	28%	28%	27%	26%	27%	26%	27%
78	95	4	15	4.31 - 4.48	34%	34%	34%	33%	34%	34%	32%	32%	32%	32%	31%	32%	32%	32%	32%	33%	32%	31%	31%	31%
96	103	1	5	7.80 - 7.99	69%	70%	72%	70%	72%	69%	71%	70%	70%	67%	70%	74%	74%	74%	74%	68%	68%	69%	69%	75%
104	117	2	11	9.82 - 10.37	23%	31%	32%	30%	31%	24%	29%	29%	30%	28%	26%	32%	31%	31%	32%	24%	30%	30%	28%	30%
118	129	2	10	6.80 - 7.02	24%	32%	33%	31%	32%	24%	30%	30%	31%	29%	25%	32%	33%	34%	34%	19%	28%	32%	32%	33%
130	155	3	24	4.59 - 4.70	21%	75%	79%	78%	79%	19%	75%	77%	79%	78%	16%	72%	78%	78%	78%	10%	56%	76%	77%	80%
130	160	4	29	5.22 - 5.91	22%	82%	87%	86%	87%	19%	83%	85%	86%	86%	17%	81%	86%	86%	87%	12%	65%	85%	86%	89%
156	172	2	14	11.08 - 11.34	48%	60%	62%	60%	61%	45%	57%	57%	59%	57%	45%	59%	60%	59%	60%	41%	54%	57%	56%	59%
156	182	3	22	10.89 - 11.05	52%	60%	61%	59%	60%	48%	55%	55%	57%	54%	49%	59%	59%	60%	59%	43%	50%	51%	52%	55%
161	172	2	9	11.59 - 11.86	55%	60%	62%	60%	61%	52%	57%	57%	59%	57%	53%	61%	61%	61%	61%	48%	55%	57%	56%	58%
161	182	2	17	11.18 - 11.39	56%	59%	61%	58%	60%	53%	54%	54%	55%	52%	55%	60%	60%	60%	61%	47%	51%	52%	51%	53%
161	182	3	17	11.20 - 11.42	57%	60%	61%	59%	60%	53%	54%	53%	55%	52%	55%	61%	60%	60%	61%	47%	51%	52%	50%	53%
161	194	4	29	10.47 - 10.68	49%	52%	53%	51%	52%	44%	46%	46%	48%	45%	46%	51%	51%	52%	52%	41%	44%	46%	46%	48%
182	194	3	11	8.24 - 8.46	40%	41%	42%	41%	42%	37%	38%	37%	39%	37%	40%	45%	44%	42%	42%	39%	40%	40%	38%	39%
183	194	2	10	7.86 - 8.12	32%	34%	35%	34%	35%	30%	33%	32%	34%	32%	31%	35%	34%	35%	35%	29%	32%	33%	33%	33%
183	194	3	10	7.88 - 8.11	32%	34%	35%	34%	35%	30%	33%	33%	34%	32%	32%	36%	35%	35%	35%	29%	33%	34%	33%	35%
195	213	3	17	3.73 - 3.85	46%	45%	46%	44%	46%	46%	44%	44%	45%	43%	45%	47%	46%	46%	46%	44%	44%	45%	44%	45%
195	213	4	17	3.69 - 3.99	45%	45%	45%	44%	45%	45%	44%	43%	45%	42%	44%	46%	45%	45%	45%	44%	43%	44%	43%	45%
195	215	2	19	4.81 - 5.01	48%	49%	49%	47%	49%	47%	46%	46%	47%	45%	47%	49%	48%	48%	49%	46%	45%	47%	46%	47%
195	215	3	19	5.02 - 5.11	49%	48%	49%	48%	49%	47%	47%	46%	48%	46%	47%	49%	48%	48%	49%	46%	46%	47%	46%	48%
195	215	4	19	4.81 - 5.18	48%	50%	49%	47%	48%	48%	47%	47%	48%	46%	47%	48%	48%	48%	48%	47%	47%	48%	46%	47%
195	219	4	23	6.53 - 6.82	56%	55%	56%	56%	55%	52%	54%	54%	55%	54%	54%	55%	55%	55%	55%	50%	52%	54%	53%	55%
195	219	5	23	6.49 - 6.66	56%	56%	57%	56%	57%	53%	55%	54%	55%	54%	55%	56%	56%	56%	57%	51%	53%	54%	54%	56%
214	229	4	14	6.81 - 6.99	46%	50%	53%	51%	52%	26%	43%	53%	53%	51%	44%	48%	50%	50%	52%	21%	31%	49%	50%	51%
216	229	3	12	4.92 - 5.42	34%	37%	41%	40%	41%	12%	28%	39%	40%	39%	33%	38%	40%	40%	40%	9%	16%	36%	39%	40%
216	229	4	12	4.92 - 5.34	34%	38%	41%	40%	41%	13%	26%	39%	40%	39%	33%	38%	40%	40%	40%	9%	16%	36%	39%	40%
216	229	5	12	4.93 - 5.34	35%	37%	41%	40%	41%	13%	29%	39%	40%	38%	32%	38%	40%	40%	40%	10%	16%	37%	39%	40%
220	229	2	8	4.58 - 4.81	38%	43%	47%	45%	47%	15%	33%	44%	46%	45%	38%	44%	47%	48%	48%	11%	19%	42%	45%	47%
220	229	4	8	4.53 - 4.81	39%	44%	47%	46%	47%	16%	33%	45%	47%	45%	38%	45%	48%	48%	49%	12%	18%	43%	45%	47%

STDEVS

Start	End	CS	CWC15 3s/4C	CWC15 3s	CWC15 30s	CWC15 300s	CWC15 3000s	CWC15/CTNNBL1 3s/4C	CWC15/CTNNBL1 3s	CWC15/CTNNBL1 30s	CWC15/CTNNBL1 300s	CWC15/CTNNBL1 3000s	CWC15/CDC5L 3s/4C	CWC15/CDC5L 3s	CWC15/CDC5L 30s	CWC15/CDC5L 300s	CWC15/CDC5L 3000s	CWC15/CDC5L/CTNNBL1 3s/4C	CWC15/CDC5L/CTNNBL1 3s	CWC15/CDC5L/CTNNBL1 30s	CWC15/CDC5L/CTNNBL1 300s	CWC15/CDC5L/CTNNBL1 3000s
75	95	3	1%	1%	1%	1%	1%	3%	1%	1%	1%	1%	0%	0%	1%	0%	1%	1%	1%	1%	1%	2%
75	95	4	1%	1%	0%	1%	1%	3%	1%	1%	2%	1%	0%	0%	1%	0%	1%	1%	2%	1%	1%	2%
75	95	5	1%	2%	1%	1%	1%	4%	1%	1%	2%	1%	0%	1%	1%	0%	1%	1%	1%	1%	1%	2%
78	95	4	1%	2%	1%	1%	2%	3%	2%	1%	1%	1%	1%	0%	1%	0%	1%	2%	1%	1%	1%	3%
96	103	1	1%	1%	1%	1%	1%	1%	1%	3%	1%	2%	2%	1%	0%	1%	1%	3%	1%	0%	1%	1%
104	117	2	1%	2%	1%	1%	2%	1%	1%	1%	1%	1%	1%	1%	1%	1%	1%	2%	1%	1%	1%	3%
118	129	2	1%	1%	1%	1%	1%	2%	1%	1%	1%	1%	1%	0%	1%	1%	1%	1%	1%	1%	1%	4%
130	155	3	1%	2%	1%	1%	1%	0%	2%	1%	1%	0%	1%	1%	1%	1%	1%	1%	2%	1%	1%	5%
130	160	4	0%	2%	0%	0%	1%	0%	1%	1%	2%	0%	1%	1%	0%	0%	0%	1%	2%	0%	1%	4%
156	172	2	1%	2%	1%	1%	2%	3%	1%	1%	2%	1%	2%	0%	1%	1%	1%	2%	2%	1%	1%	3%
156	182	3	1%	2%	2%	1%	2%	2%	2%	2%	2%	1%	1%	1%	1%	1%	2%	3%	2%	1%	0%	3%
161	172	2	3%	2%	1%	2%	1%	2%	2%	1%	2%	1%	2%	1%	1%	1%	1%	2%	2%	1%	1%	4%
161	182	2	1%	2%	2%	1%	2%	3%	2%	2%	2%	2%	2%	1%	1%	1%	1%	2%	2%	2%	2%	5%
161	182	3	1%	2%	2%	1%	2%	4%	2%	2%	2%	2%	2%	1%	1%	1%	1%	2%	3%	2%	2%	5%
161	194	4	1%	2%	1%	1%	2%	2%	2%	1%	2%	2%	2%	1%	1%	1%	1%	2%	2%	2%	1%	3%
182	194	3	0%	1%	1%	0%	1%	2%	1%	1%	1%	1%	1%	2%	2%	1%	1%	3%	1%	1%	1%	3%
183	194	2	1%	1%	1%	1%	1%	2%	1%	1%	1%	0%	1%	1%	1%	1%	1%	1%	2%	1%	1%	3%
183	194	3	1%	1%	1%	1%	1%	2%	1%	1%	1%	1%	1%	1%	1%	1%	1%	1%	2%	1%	1%	2%
195	213	3	1%	2%	1%	1%	1%	3%	2%	1%	1%	1%	1%	1%	1%	1%	1%	1%	2%	1%	1%	4%
195	213	4	1%	2%	1%	1%	1%	3%	2%	1%	2%	1%	1%	1%	1%	1%	1%	1%	2%	1%	1%	4%
195	215	2	1%	2%	2%	1%	2%	1%	2%	1%	1%	2%	2%	1%	1%	1%	2%	1%	2%	2%	1%	4%
195	215	3	1%	2%	1%	1%	1%	2%	1%	1%	2%	1%	1%	0%	1%	1%	1%	1%	2%	1%	1%	4%
195	215	4	1%	1%	1%	1%	2%	2%	1%	1%	1%	1%	2%	1%	1%	1%	1%	1%	2%	1%	1%	5%
195	219	4	0%	2%	1%	0%	1%	1%	1%	1%	1%	0%	1%	1%	0%	0%	1%	1%	2%	0%	1%	4%
195	219	5	0%	1%	1%	0%	1%	1%	1%	1%	1%	1%	1%	1%	1%	0%	1%	1%	2%	0%	1%	4%
214	229	4	1%	2%	1%	1%	1%	1%	0%	2%	1%	1%	1%	1%	1%	1%	1%	0%	1%	1%	2%	3%
216	229	3	0%	1%	1%	0%	1%	2%	1%	1%	1%	1%	1%	0%	1%	1%	0%	0%	1%	0%	1%	3%
216	229	4	1%	1%	1%	0%	1%	1%	1%	1%	1%	0%	1%	0%	1%	0%	0%	2%	0%	0%	1%	2%
216	229	5	0%	0%	1%	0%	2%	2%	1%	1%	1%	2%	2%	1%	1%	1%	1%	2%	1%	0%	1%	2%
220	229	2	1%	2%	1%	1%	1%	1%	1%	1%	2%	1%	1%	0%	1%	1%	1%	0%	1%	1%	1%	4%
220	229	4	1%	2%	1%	1%	1%	1%	1%	1%	1%	1%	1%	1%	1%	1%	1%	1%	1%	1%	1%	3%

Relative deuteration %

10%	20%	30%	40%	50%	60%	70%	80%

Fig. S5-C

Global HDX levels

Start	End	CS	#D	RT	CDC5L 3s/4C	CDC5L 3s	CDC5L 30s	CDC5L 300s	CDC5L 3000s	CDC5L/CWC15 3s/4C	CDC5L/CWC15 3s	CDC5L/CWC15 30s	CDC5L/CWC15 300s	CDC5L/CWC15 3000s	CDC5L/CWC15/CTNNBL1 3s/4C	CDC5L/CWC15/CTNNBL1 3s	CDC5L/CWC15/CTNNBL1 30s	CDC5L/CWC15/CTNNBL1 300s	CDC5L/CWC15/CTNNBL1 3000s
11	19	2	7	8.83 - 9.10	3%	9%	24%	35%	37%	4%	9%	22%	37%	39%	6%	8%	17%	29%	37%
18	24	2	5	6.68 - 6.91	0%	9%	62%	80%	82%	1%	6%	45%	82%	83%	3%	6%	25%	63%	71%
20	37	4	16	7.72 - 8.05	19%	31%	58%	61%	62%	18%	28%	50%	63%	65%	13%	24%	41%	59%	66%
20	38	4	17	9.42 - 9.75	19%	30%	59%	64%	65%	17%	26%	49%	65%	66%	12%	22%	40%	60%	68%
22	37	3	14	8.32 - 8.63	20%	31%	56%	59%	60%	18%	28%	49%	62%	62%	13%	23%	40%	57%	63%
22	37	4	14	8.34 - 8.65	20%	31%	56%	59%	60%	18%	27%	49%	61%	62%	13%	23%	40%	57%	63%
22	38	3	15	9.93 - 10.20	20%	30%	58%	61%	63%	17%	26%	48%	63%	64%	12%	22%	38%	57%	61%
22	50	6	27	7.61 - 7.79	10%	17%	36%	37%	39%	12%	17%	34%	43%	44%	7%	12%	25%	37%	42%
24	37	3	12	8.28 - 8.59	22%	34%	62%	63%	65%	20%	30%	53%	67%	67%	14%	25%	42%	63%	68%
25	37	2	11	7.87 - 8.23	20%	29%	53%	55%	57%	20%	29%	45%	58%	59%	13%	23%	35%	52%	58%
25	37	3	11	7.93 - 8.19	20%	29%	52%	54%	56%	18%	27%	43%	56%	56%	13%	22%	34%	51%	57%
38	50	2	11	3.48 - 3.74	2%	11%	26%	26%	27%	2%	8%	24%	27%	28%	1%	3%	15%	25%	27%
38	50	3	11	3.48 - 3.70	2%	11%	26%	26%	27%	2%	8%	24%	28%	27%	1%	3%	15%	25%	27%
38	50	4	11	3.49 - 3.77	2%	11%	26%	26%	27%	2%	8%	25%	28%	28%	2%	3%	15%	26%	27%
38	50	5	11	3.56 - 3.73	2%	12%	26%	26%	27%	2%	8%	25%	28%	28%	1%	3%	15%	26%	27%
38	71	6	31	8.08 - 8.33	11%	19%	37%	41%	42%	11%	19%	34%	44%	45%	10%	15%	28%	40%	43%
39	50	4	10	3.48 - 3.72	2%	12%	28%	28%	29%	2%	8%	26%	30%	30%	2%	3%	16%	28%	29%
51	66	4	13	8.62 - 8.82	19%	26%	34%	34%	35%	20%	29%	37%	40%	40%	17%	25%	33%	35%	37%
51	71	4	18	8.59 - 8.83	12%	20%	35%	40%	41%	13%	22%	35%	46%	46%	12%	19%	32%	42%	45%
51	74	4	21	9.42 - 9.73	12%	21%	44%	52%	53%	13%	22%	41%	55%	56%	12%	19%	36%	51%	54%
51	86	6	32	10.07 - 10.26	16%	29%	56%	62%	63%	15%	27%	52%	65%	65%	13%	22%	46%	61%	65%
51	96	6	41	10.32 - 10.54	18%	39%	65%	71%	72%	17%	35%	61%	71%	72%	15%	30%	57%	69%	73%
51	98	6	43	10.78 - 11.02	17%	38%	66%	71%	73%	15%	34%	62%	73%	73%	13%	30%	59%	71%	74%
72	86	3	12	8.88 - 9.15	17%	34%	55%	54%	56%	18%	33%	56%	60%	61%	13%	25%	51%	56%	58%
75	86	3	9	8.16 - 8.45	23%	42%	58%	58%	61%	34%	46%	60%	63%	64%	24%	36%	55%	57%	60%
75	96	3	18	9.03 - 9.26	21%	53%	72%	71%	73%	19%	46%	73%	75%	76%	17%	41%	70%	73%	75%
87	96	2	8	5.80 - 6.23	23%	60%	75%	74%	76%	20%	53%	73%	75%	77%	18%	46%	70%	71%	73%
87	98	2	10	7.97 - 8.26	16%	45%	64%	63%	66%	15%	40%	66%	67%	69%	14%	39%	67%	67%	69%
96	103	2	6	10.20 - 10.47	1%	8%	29%	30%	30%	1%	6%	28%	33%	33%	2%	6%	29%	31%	32%
97	102	2	4	8.59 - 8.88	1%	12%	42%	42%	43%	1%	9%	39%	44%	44%	2%	9%	37%	38%	40%
97	103	2	5	10.00 - 10.29	2%	8%	30%	30%	31%	1%	6%	28%	33%	34%	2%	6%	26%	28%	30%
98	104	2	5	10.00 - 10.29	1%	8%	30%	30%	31%	1%	6%	28%	33%	33%	1%	6%	26%	28%	29%
102	113	3	10	7.80 - 8.14	20%	28%	46%	49%	50%	18%	24%	39%	50%	49%	12%	20%	30%	43%	50%
103	111	2	7	5.85 - 6.19	27%	67%	85%	84%	87%	23%	61%	84%	86%	88%	21%	53%	80%	81%	84%
103	113	2	9	4.04 - 4.21	51%	57%	58%	57%	58%	48%	58%	58%	58%	58%	45%	54%	56%	55%	57%
103	113	3	9	4.04 - 4.26	52%	59%	59%	58%	59%	49%	59%	59%	59%	60%	46%	55%	57%	56%	58%
103	144	5	33	6.69 - 6.77	38%	41%	41%	39%	41%	40%	45%	45%	45%	45%	36%	40%	41%	40%	42%
103	144	6	33	6.66 - 7.04	38%	41%	40%	39%	40%	40%	45%	44%	44%	44%	36%	40%	41%	40%	42%
103	146	6	35	6.67 - 6.81	34%	37%	37%	36%	37%	37%	42%	41%	41%	41%	33%	37%	38%	37%	39%
104	113	2	8	4.03 - 4.21	54%	61%	61%	60%	62%	50%	61%	61%	61%	61%	48%	58%	59%	58%	60%
104	144	5	32	6.54 - 6.76	37%	40%	40%	39%	40%	40%	45%	45%	45%	45%	35%	39%	40%	39%	41%
105	113	2	7	4.03 - 4.21	59%	67%	68%	66%	68%	56%	68%	68%	68%	69%	53%	63%	65%	64%	66%
114	143	4	21	6.01 - 6.30	43%	45%	46%	45%	46%	46%	50%	50%	50%	50%	41%	44%	45%	45%	47%
114	144	3	22	6.91 - 6.99	41%	44%	44%	42%	44%	44%	48%	48%	48%	48%	38%	42%	43%	43%	45%
114	144	5	22	6.86 - 7.02	42%	44%	44%	43%	44%	44%	48%	48%	47%	48%	39%	43%	44%	43%	45%
150	195	6	44	4.96 - 5.62	47%	58%	58%	58%	60%	36%	59%	59%	60%	60%	14%	24%	50%	58%	60%
179	195	4	15	3.96 - 4.18	41%	50%	50%	49%	51%	40%	52%	51%	51%	52%	27%	34%	45%	48%	50%

STDEVS

Start	End	CS	CDC5L 3s/4C	CDC5L 3s	CDC5L 30s	CDC5L 300s	CDC5L 3000s	CDC5L/CWC15 3s/4C	CDC5L/CWC15 3s	CDC5L/CWC15 30s	CDC5L/CWC15 300s	CDC5L/CWC15 3000s	CDC5L/CWC15/CTNNBL1 3s/4C	CDC5L/CWC15/CTNNBL1 3s	CDC5L/CWC15/CTNNBL1 30s	CDC5L/CWC15/CTNNBL1 300s	CDC5L/CWC15/CTNNBL1 3000s
11	19	2	0	1	1	1	1	0	0	2	1	1	0	1	1	1	4
18	24	2	0	1	1	1	2	1	1	3	2	1	1	2	3	0	n/a
20	37	4	1	2	1	2	1	0	1	2	1	0	0	2	1	1	4
20	38	4	0	1	1	0	0	1	0	1	0	1	0	1	1	1	4
22	37	3	1	2	1	1	1	1	0	2	0	1	0	1	1	1	4
22	37	4	1	1	2	1	2	1	0	2	1	1	0	1	1	1	4
22	38	3	1	2	1	1	1	1	0	2	0	1	1	1	1	1	1
22	50	6	0	1	1	0	1	1	1	1	0	1	0	1	0	1	4
24	37	3	1	2	1	0	1	0	1	2	0	1	0	1	0	1	4
25	37	2	1	1	1	1	2	1	0	1	1	0	0	1	1	1	4
25	37	3	1	1	1	1	0	1	0	2	1	0	0	1	0	1	3
38	50	2	0	1	0	0	0	0	0	1	0	1	0	0	0	1	2
38	50	3	0	1	0	0	0	0	0	1	0	1	0	0	1	1	2
38	50	4	0	1	0	0	0	0	0	1	1	1	0	0	0	1	2
38	50	5	0	1	0	0	1	0	0	1	0	1	0	0	1	1	2
38	71	6	1	1	1	1	1	0	0	2	1	0	1	1	1	1	3
39	50	4	0	1	1	0	0	0	0	1	1	1	0	1	0	1	3
51	66	4	1	2	1	0	1	1	1	1	1	1	1	2	1	1	3
51	71	4	1	1	1	1	1	0	0	2	1	1	1	1	1	1	3
51	74	4	1	2	1	1	1	1	0	3	1	1	1	1	1	1	3
51	86	6	1	2	1	1	1	1	0	2	1	1	1	1	1	1	3
51	96	6	1	2	0	1	1	1	0	2	1	1	1	1	1	0	3
51	98	6	1	2	0	1	1	1	0	2	1	1	1	1	1	0	3
72	86	3	1	2	1	1	1	1	0	2	1	1	0	2	1	1	4
75	86	3	1	2	0	1	3	1	1	2	1	2	1	1	2	1	2
75	96	3	0	2	0	1	1	1	0	1	1	1	1	2	0	1	4
87	96	2	0	2	1	1	1	1	1	2	1	1	1	2	1	1	5
87	98	2	0	3	2	2	1	1	0	2	1	1	1	2	1	1	4
96	103	2	0	1	1	1	1	0	0	1	0	1	0	1	1	1	3
97	102	2	0	1	0	1	1	0	0	2	1	0	0	1	1	1	4
97	103	2	0	1	0	0	1	0	0	1	1	1	0	1	1	1	3
98	104	2	0	1	1	1	1	0	0	1	1	1	0	1	1	1	3
102	113	3	1	1	0	1	1	1	0	2	0	0	0	1	0	1	1
103	111	2	1	0	1	2	2	1	0	2	1	1	1	2	2	1	7
103	113	2	2	2	1	1	1	2	0	1	1	1	1	2	1	2	5
103	113	3	2	2	1	1	1	2	1	1	1	1	1	2	1	1	4
103	144	5	2	2	1	1	1	1	1	1	1	1	1	2	1	1	4
103	144	6	2	1	1	0	1	1	1	1	1	1	1	2	1	1	4
103	146	6	2	2	1	1	1	1	0	1	1	1	1	2	1	1	4
104	113	2	2	2	1	1	1	2	1	2	1	1	1	2	1	1	5
104	144	5	2	2	0	1	0	1	1	1	1	1	1	2	1	1	4
105	113	2	2	2	1	1	1	2	1	1	1	1	1	3	1	2	5
114	143	4	2	1	0	0	0	1	1	1	0	1	2	2	0	1	4
114	144	3	2	2	1	1	1	1	1	1	1	1	1	2	1	1	4
114	144	5	2	2	0	0	1	1	1	1	1	1	1	2	1	1	4
150	195	6	0	1	0	0	1	1	1	1	1	1	1	0	1	1	4
179	195	4	1	2	1	1	1	2	1	1	1	1	1	1	1	1	4

Relative deuteration %	10%	20%	30%	40%	50%	60%	70%	80%

Fig. S6

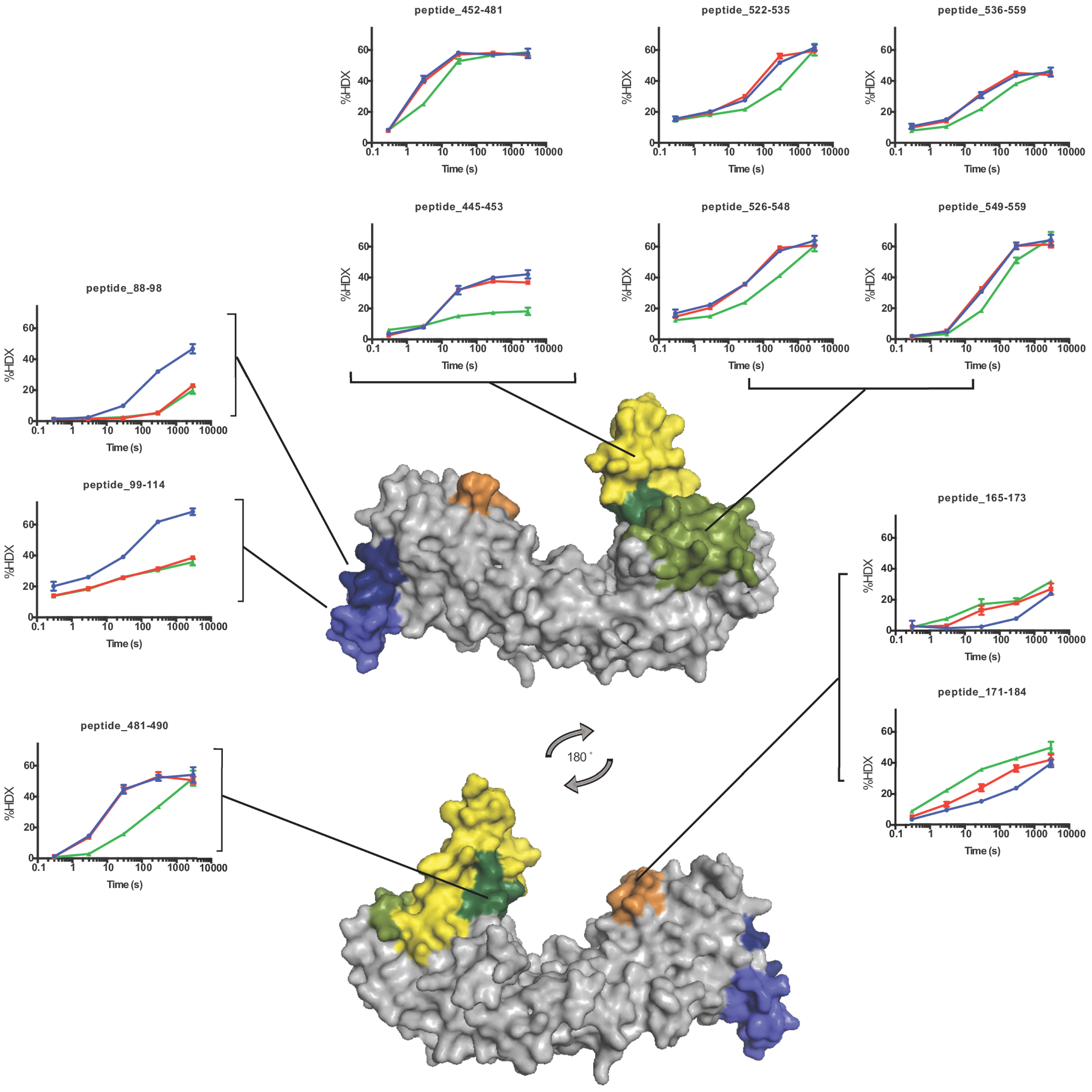

peptide_452-481
peptide_522-535
peptide_536-559
peptide_445-453
peptide_526-548
peptide_549-559
peptide_88-98
peptide_99-114
peptide_165-173
peptide_171-184
peptide_481-490
%HDX
Time (s)
0.1
1
10
100
1000
10000
180 °

Fig. S7

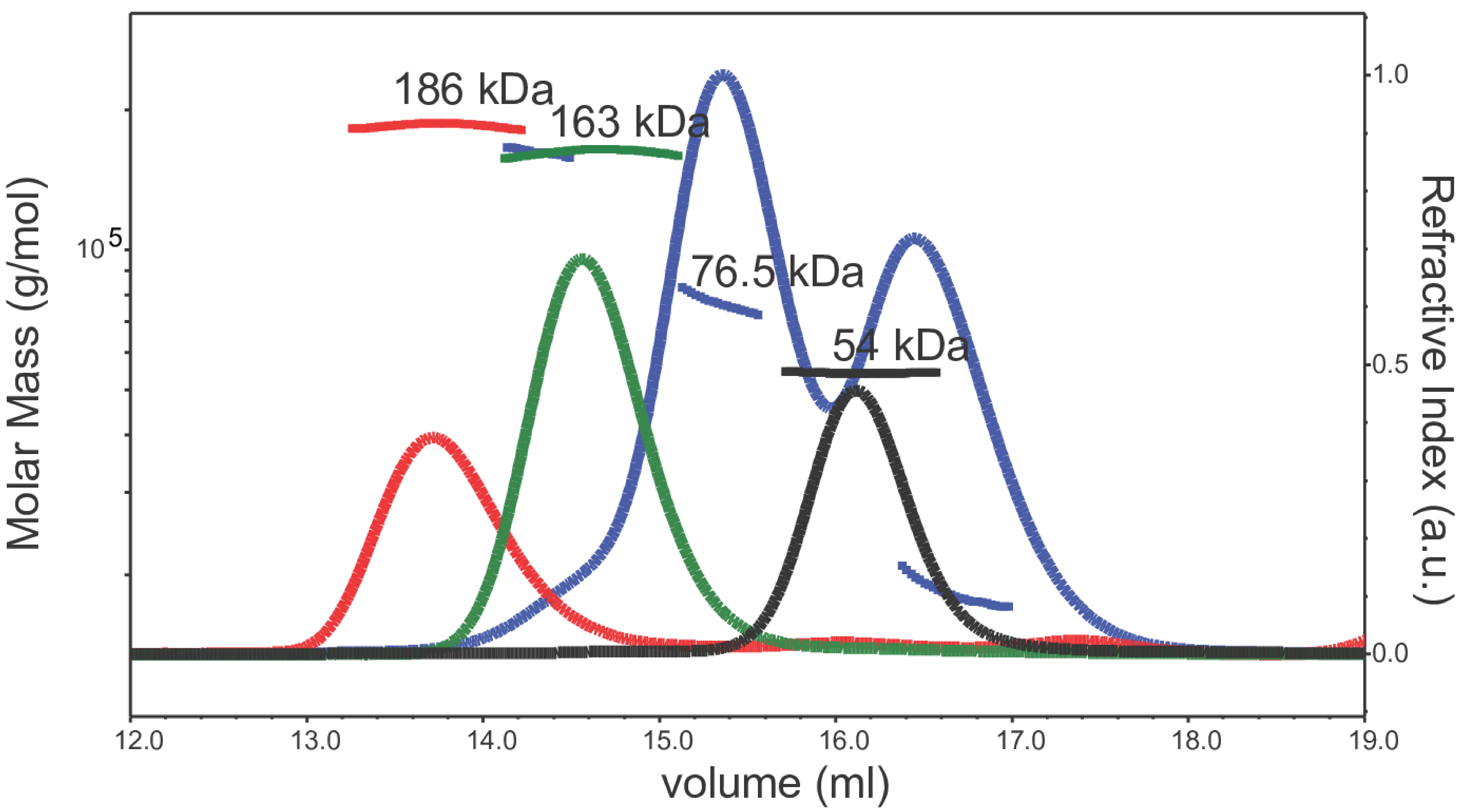

186 kDa
163 kDa
76.5 kDa
54 kDa
Molar Mass (g/mol)
10^5
Refractive Index (a.u.)
1.0
0.5
0.0
12.0
13.0
14.0
15.0
16.0
17.0
18.0
19.0
volume (ml)

Fig. S8

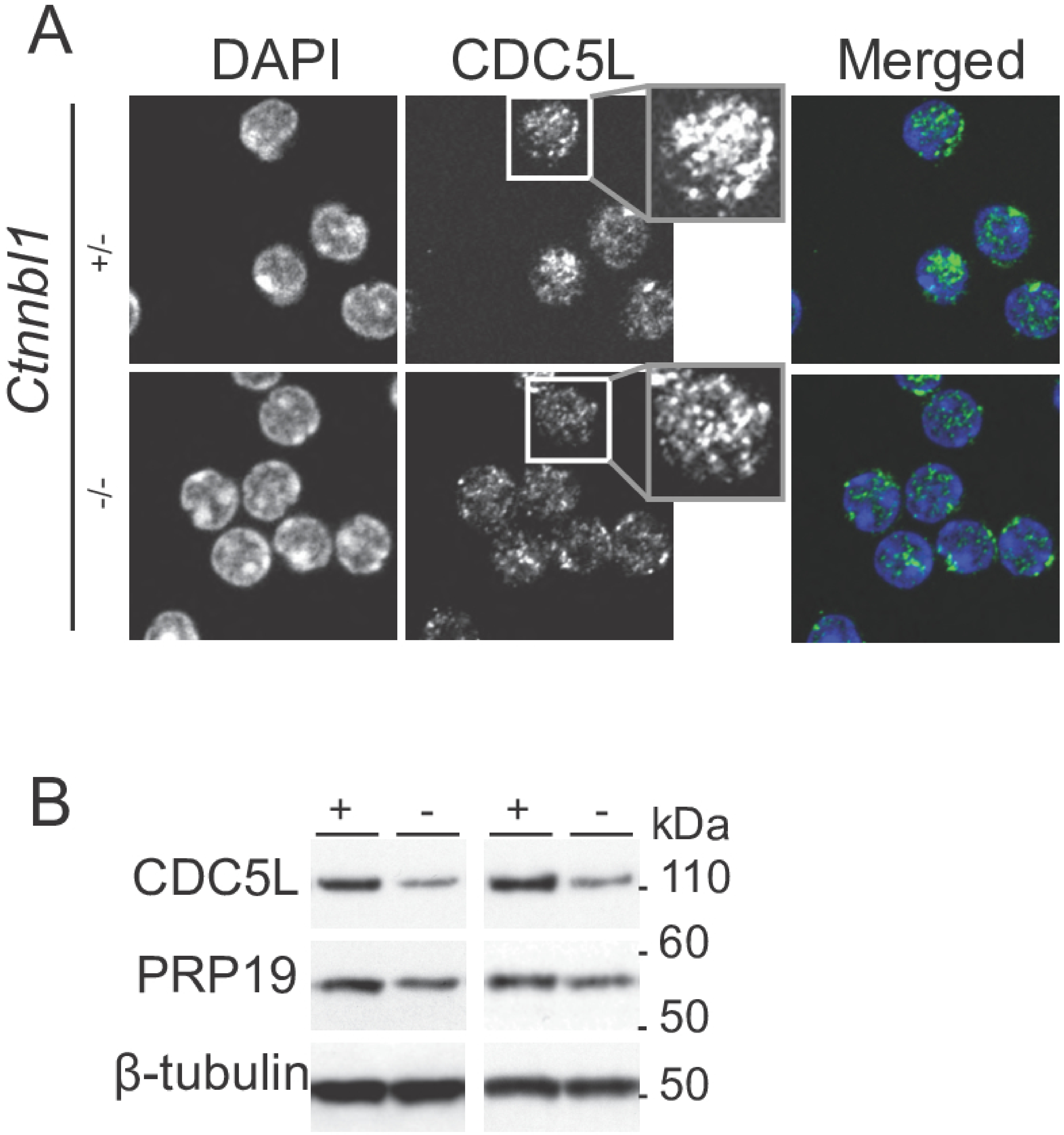
A
DAPI
CDC5L
Merged
Ctnnbl1
+/-
-/-
B
+
-
+
-
kDa
CDC5L
110
PRP19
60
50
β-tubulin
50

Fig. S9

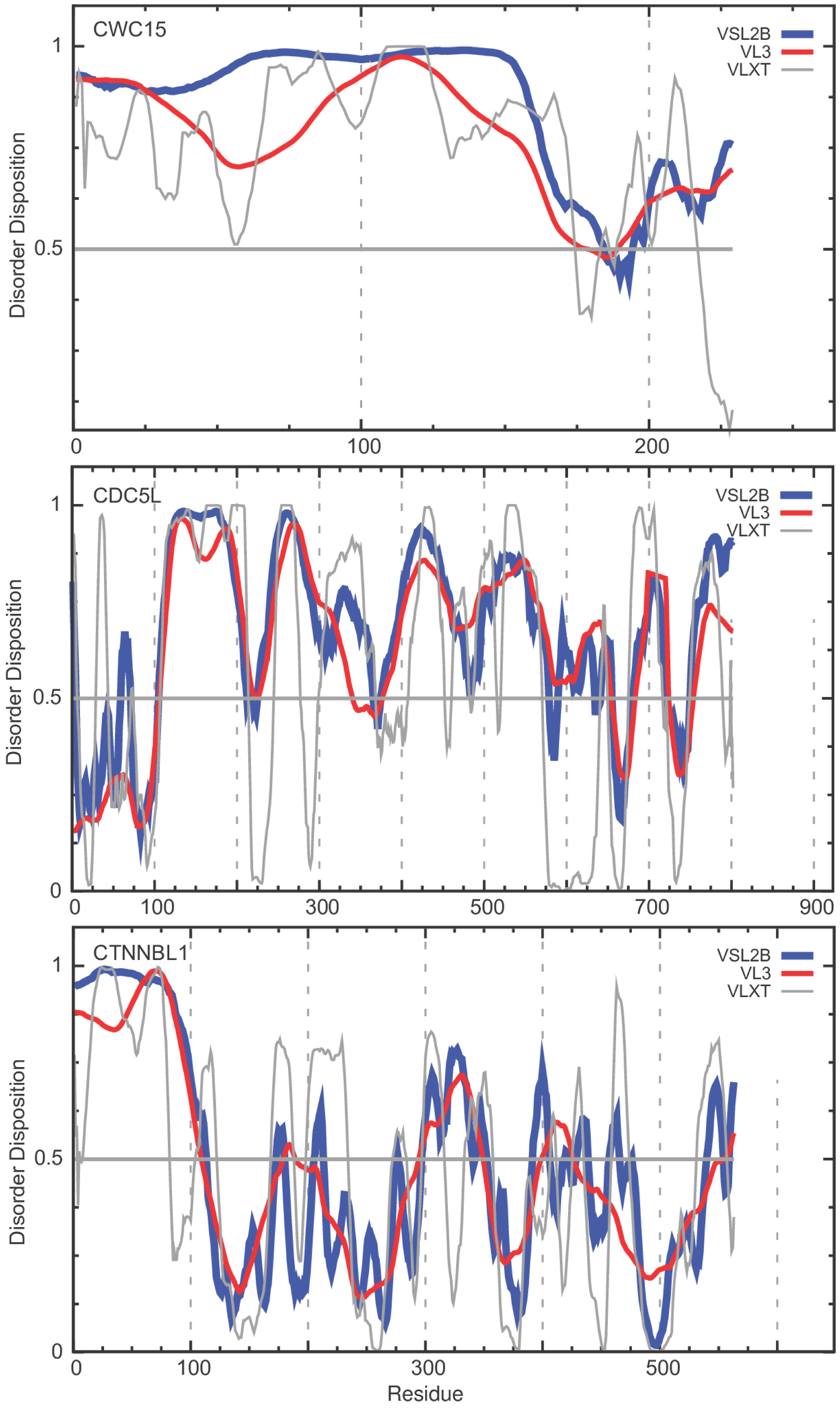

CWC15
CDC5L
CTNNBL1
VSL2B
VL3
VLXT
Disorder Disposition
Residue
0
0.5
1
100
200
300
500
700
900
